# An Innovative Strategy for Untargeted Mass Spectrometry Data Analysis: Rapid Chemical Profiling of the Medicinal Plant *Terminalia chebula* Using Ultra-High-Performance Liquid Chromatography Coupled with Q/TOF Mass Spectrometry–Key Ion Diagnostics–Neutral Loss Filtering

**DOI:** 10.3390/molecules30112451

**Published:** 2025-06-03

**Authors:** Jia Yu, Xinyan Zhao, Yuqi He, Yi Zhang, Ce Tang

**Affiliations:** 1 School of Ethnic Medicine, Chengdu University of Traditional Chinese Medicine, Chengdu 611137, China; yu77@cdutcm.edu.cn; 2 School of Pharmacy, Chengdu University of Traditional Chinese Medicine, Chengdu 611137, China; 19946734998@163.com (X.Z.); 18502828016@163.com (Y.H.)

**Keywords:** *Terminalia chebula*, key ion diagnostics, neutral loss filtering, untargeted mass spectrometry, UPLC-Q-TOF/MS

## Abstract

Structural characterization of natural products in complex herbal extracts remains a major challenge in phytochemical analysis. In this study, we present a novel post-acquisition data-processing strategy—key ion diagnostics–neutral loss filtering (KID-NLF)—combined with ultra-high-performance liquid chromatography–quadrupole time-of-flight mass spectrometry (UPLC-Q/TOF-MS) for systematic profiling of the medicinal plant *Terminalia chebula*. The strategy consists of four main steps. First, untargeted data are acquired in negative electrospray ionization (ESI^−^) mode. Second, a genus-specific diagnostic ion database is constructed by leveraging characteristic fragment ions (e.g., gallic acid, chebuloyl, and HHDP groups) and conserved substructures. Third, MS/MS data are high-resolution filtered using key ion diagnostics and neutral loss patterns (302 Da for HHDP; 320 Da for chebuloyl). Finally, structures are elucidated via detailed spectral analysis. The methanol extract of *T. chebula* was separated on a C18 column using a gradient of acetonitrile and 0.1% aqueous formic acid within 33 min. This separation enabled detection of 164 compounds, of which 47 were reported for the first time. Based on fragmentation pathways and diagnostic ions (e.g., *m*/*z* 169 for gallic acid, *m*/*z* 301 for ellagic acid, and neutral losses of 152, 302, and 320 Da), the compounds were classified into three major groups: gallic acid derivatives, ellagitannins (containing HHDP, chebuloyl, or neochebuloyl moieties), and triterpenoid glycosides. KID-NLF overcomes key limitations of conventional workflows—namely, isomer discrimination and detection of low-abundance compounds—by exploiting genus-specific structural signatures. This strategy demonstrates high efficiency in resolving complex polyphenolic and triterpenoid profiles and enables rapid annotation of both known and novel metabolites. This study highlights KID-NLF as a robust framework for phytochemical analysis in species with high chemical complexity. It also paves the way for applications in quality control, drug discovery, and mechanistic studies of medicinal plants.

## 1. Introduction

The exploration of medicinal plants has gained significant momentum globally, driven by their long-established therapeutic history and untapped potential for drug discovery. A central aspect of this effort is the thorough analysis of the chemical composition of medicinal plants, as their efficacy, safety, and quality are inherently tied to their phytochemical profiles [1]. High-resolution mass spectrometry (HRMS), particularly when combined with ultra-high-performance liquid chromatography (UPLC), has become a cornerstone technology for analyzing complex plant extracts, facilitating the rapid identification of both known and novel compounds [2]. However, some limitations remain. For instance, the peak capacity is limited, making it challenging to fulfill the separation requirements of chemical components in the complex systems of medicinal plants [3]. Traditional identification strategies, which rely on chromatographic retention behavior and mass spectrometry cleavage rules, are constrained by a limited number of reference substances and inadequate structural coverage in dedicated databases [4].

Current strategies for LC-MS/MS data processing are typically classified into targeted and untargeted approaches [5]. Targeted methods rely on predefined compound libraries and fragmentation pathways, offering high specificity but limited capacity for discovering novel compounds. In contrast, untargeted methods capture MS/MS spectra for all detectable ions, providing a comprehensive chemical profile but resulting in large datasets that require advanced post-acquisition processing. In recent years, feature-based molecular networking (FBMN) [6] and ion identity molecular networking (IIMN) have enhanced metabolite grouping by integrating retention time and admixture data [7]. Additionally, tools such as SIRIUS 5, powered by deep learning, have increased annotation accuracy through fragment tree calculations [8]. However, these general strategies remain inadequate for highly specific structures. These include excimer ion matching errors, automatic matching of endogenous cleavage fragments to other components, and the low recognition accuracy of complex additive ions [9].

*Terminalia chebula*, known as the “king of Tibetan medicine”, has been used for thousands of years in traditional Chinese and Ayurvedic medicine [10]. *T. chebula* is used in the treatment of asthma, bronchitis, hepatitis, dyspepsia, eye diseases, and hoarseness and to promote hair growth [11]. The flesh of the plant has been used to treat diarrhea, leprosy, and edema [12]. It improves appetite, reduces cholesterol and blood pressure, strengthens the immune system, prevents aging, and enhances resistance to infections [13]. In clinical practice, the therapeutic effects of specific preparations can be tailored to treat various diseases and optimized by combining them based on distinct cold and heat symptoms [14]. *T. chebula* contains a diverse array of chemical constituents, primarily including phenolic acids, tannins, triterpenoids, and flavonoids [15]. In *T. chebula*, 33% of the total phytoconstituents are hydrolysable tannins, with variation between 20 and 50%. These tannins contain phenolic carboxylic acids such as gallic acid, as well as gallotannins. Ellagitannins including punacalagin, casuarinin, corilagin, and terchebulin as well as others such as chebulanin, neochebulinic, chebulagic, and chebulinic acids are also present [16]. However, the chemical complexity of *T. chebula* poses a unique analytical challenge. Its ellagic tannins often contain multiple configurations, including HHDP, chebulyl, or neochebulyl groups. Numerous isomers with the same molecular weight exist, and their fragment pathways are complex. Most components still lack systematic annotation, which significantly hinders the establishment of quality control standards and the investigation of the material basis for its efficacy. Therefore, addressing the challenge of component analysis in *T. chebula* can reveal the chemical nature of its therapeutic potential and offer insights into the metabolomic analysis of similar complex plants.

To overcome the above limitations, combined with the additive ions that are easily formed by the *T. chebula* components and the characteristic fragments, key ion diagnostics–neutral loss filtering (KID-NLF) is presented: a post-acquisition data processing approach that leverages characteristic fragment ions and neutral loss patterns of target compounds. KID-NLF constructs a genus-specific diagnostic ion database based on known *T. chebula* metabolites and their fragmentation profiles. This enables the prioritization of structurally relevant ions in complex, untargeted MS/MS data, thereby improving both the speed and accuracy of compound identification. The approach consists of two key components: (1) key ion diagnosis (KID), which utilizes high-resolution MS/MS data to identify diagnostic fragment ions specific to gallic acid derivatives, ellagitannins, and triterpenoids; and (2) neutral loss filtering (NLF), which systematically detects characteristic mass losses (e.g., 302 Da for HHDP, 320 Da for chebuloyl) to isolate structurally related compounds. This approach addresses challenges in isomer discrimination and low-abundance compound detection by exploiting the unique structural signatures of *T. chebula* constituents. It offers a robust framework for profiling even the most complex plant extracts.

In this study, KID-NLF was applied to analyze methanol extracts of *T. chebula* using UPLC-Q/TOF-MS. The method enabled the rapid identification of 164 compounds, 47 of which are newly reported. KID-NLF overcomes limitations of existing workflows by combining targeted fragmentation analysis with genus-specific database curation. This provides a transformative strategy for phytochemical profiling, especially in species rich in polyphenols and triterpenoids. The strategy enhances understanding of *T. chebula*’s chemical diversity and provides a scalable framework for quality control and drug discovery in medicinal plant research.

## 2. Results and Discussion

### 2.1. Research Strategy

**Figure 1:**
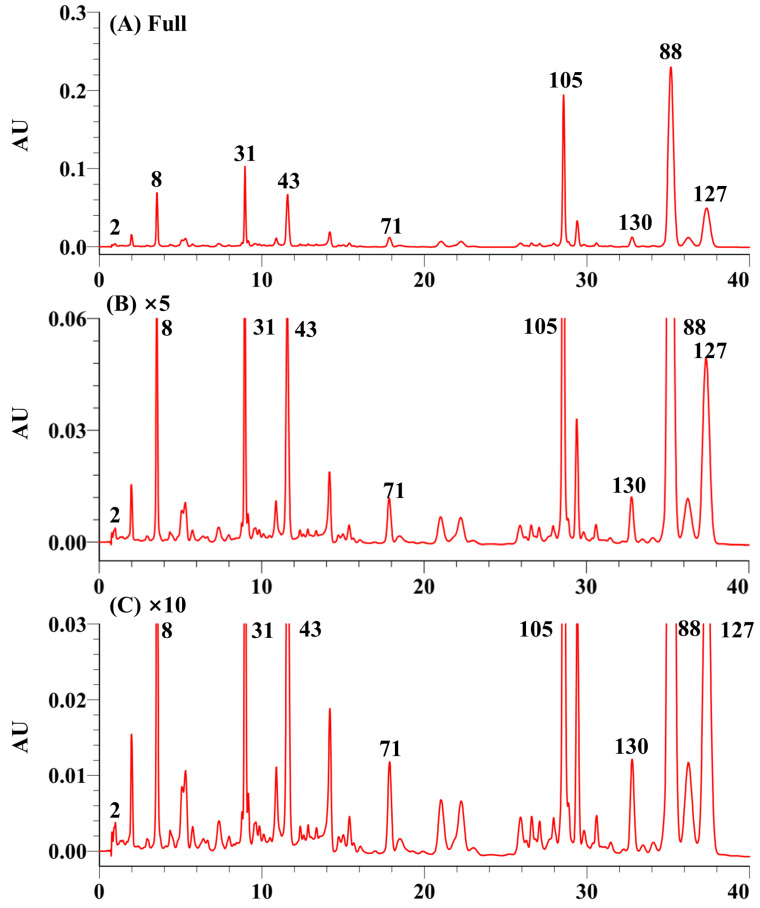
UHPLC/UV chromatograms of *Terminalia chebula* extract (254 nm).

Despite observing only a few major peaks in the UHPLC/UV analysis of *T. chebula*, the enlarged chromatogram revealed numerous minor compounds (Figure 1). To conduct a comprehensive analysis of these compounds, a 70% methanol extract was examined using UPLC-Q-TOF/MS in a non-targeted method (without specifying the parent ion). Operating in (—)-ESI mode, the analysis detected numerous compounds, facilitating the acquisition of key quasi-molecular ions with enhanced sensitivity. During the scan, all parent ions underwent analysis in MS^E^ mode at a collision energy ranging from 20 to 40 eV. Using these settings, satisfactory secondary mass spectrometry data were obtained.

Subsequently, the data underwent processing using targeted KID-NLF efficiently identifying the parent ion and its respective daughter ion structure. The fundamental structure of *T. chebula* compounds, gallic acid, features similar skeletons and substitution patterns that facilitate the generation of KID and NLF in MS/MS spectra. KID-NLF were identified from the MS/MS spectra of reference substances, leading to the creation of a database cataloguing ions related to all reported *Terminalia* compound structures. High-resolution KID were next extracted via UNIFI software (Version 1.9) for MS diagnosis and MS/MS spectral data filtering, achieving rapid and efficient structural identification. Ultimately, further determination of the identified compounds’ structures was accomplished through in-depth analysis of their high-resolution MS and MS/MS spectra.

**Figure 2:**
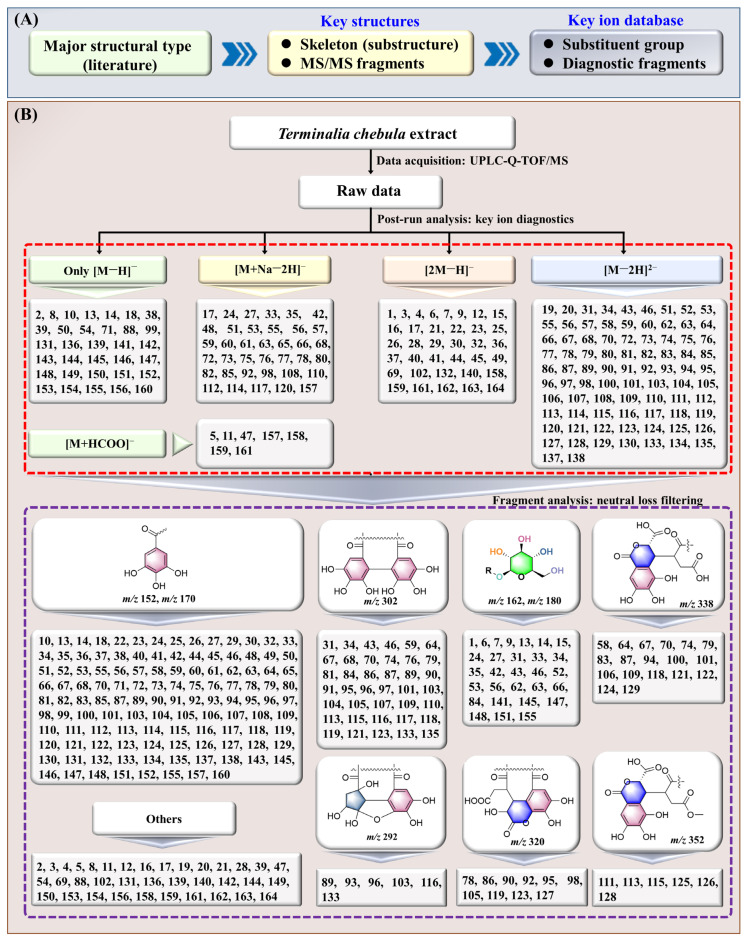
Construction of key ion database (**A**) and the identification of 164 chemical constituents in *T. chebula* using the KID-NLF strategy (**B**). The red dashed box marks components compliant with the KID strategy, the purple dashed box denotes those not only adhering to the KID strategy but also aligning with the NLF rules.

**Figure 3:**
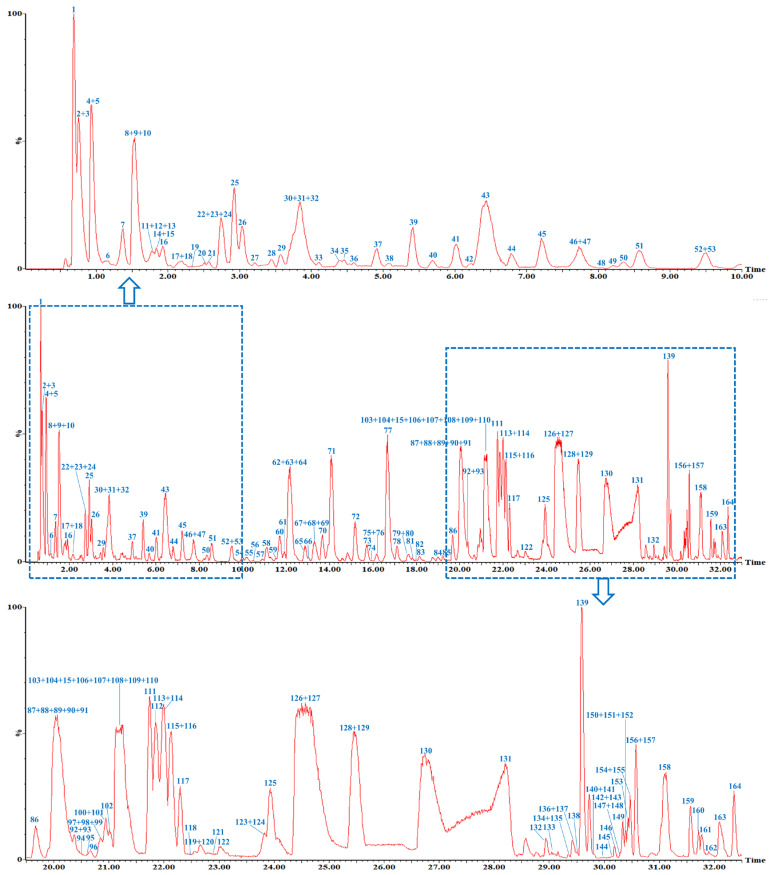
The representative base peak intensity (BPI) chromatogram of *T. chebula* in negative ion mode. The box and arrow denote the enlarged section.

In summary, the analytical strategy comprises four key steps: (1) acquisition of high-resolution MS and MS/MS data via UPLC-Q-TOF/MS; (2) creation of a diagnostic ion database informed by MS/MS fragments, similar skeletons, and substitution patterns of reported *T. chebula* compounds; (3) identification of precursor ions and significant fragments using high-resolution diagnostic ions and neutral loss filtering; and (4) further elucidation of target compound structures through detailed MS and MS/MS spectral analysis (Figure 2). According to the KID-NLF strategy, a total of 164 components were identified (Figure 3).

### 2.2. Establishing a Diagnostic Ion Database

The process for establishing the database is illustrated in Figure 2A. Initially, the literature was reviewed to summarize and classify *T. chebula* compounds into three main structural types: gallic acid and its derivatives, ellagic acid and its derivatives, and terpenoids. Subsequently, characteristic diagnostic ions were identified for each type of compound.

For gallic acid and its derivatives, diagnosis involved identifying quasi-molecular ions, including [M–2H]^2−^ and [M–H]^−^ ions. These compounds frequently feature multiple galloyl groups or exhibit neutral loss of gallic acid, resulting in ions such as [M–H–152]^−^ and [M–H–170]^−^. Consequently, the accurate masses of [M–H–152]^−^, [M–H–170]^−^, and potential derivatives were utilized to develop a diagnostic ion database.

**Figure 4:**
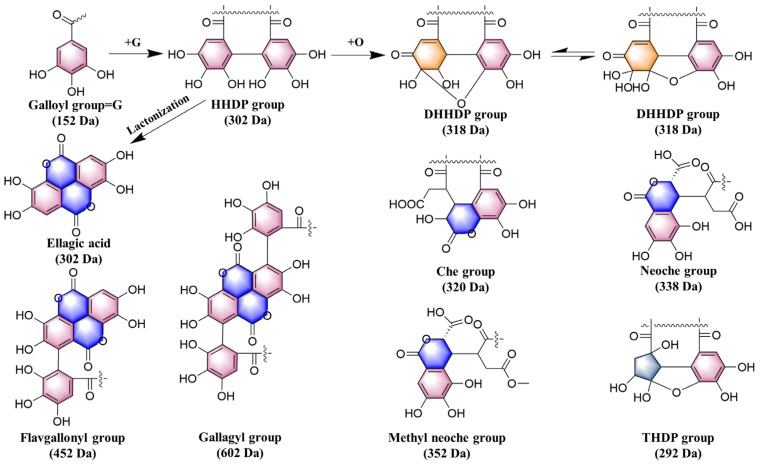
The main neutral loss fragment in *T. chebula*.

*T. chebula* contains ellagic acid and its derivatives, typically comprising HHDP (302 Da), chebuloyl (320 Da), neoche (338 Da), THDP (292 Da), DHHDP (318 Da), flavgallonyl (452 Da), and Gallagyl (602 Da) groups. These groups form complex combinations, possibly including HHDP alongside groups such as chebuloyl, neoche, THDP, DHHDP, Gallagyl, or flavgallonyl (Figure 4). In a similar manner, a diagnostic ion database for *T. chebula* ellagic acid compounds was created using the accurate masses of potential derivatives.

Terpenoids typically yield abundant deprotonated molecular ions [M–H]^−^ in the primary mass spectrometer, with some compounds also producing [2M–H]^−^ ions. Terpenoids in *T. chebula* commonly attach to sugar groups like glucose (Glc), galloyl, and glucoheptonic acid via various substitution methods. Aglycone fragments, which are relatively stable, typically lose sugar and galloyl segments (152 Da, 162 Da, 208 Da) under standard voltage. Analysis of terpenoid cleavage fragments reveals two primary types of aglycones, 503 Da and 487 Da, each with various configurations. Ultimately, the diagnostic ion database for *T. chebula* terpenoids was developed using the accurate masses of potential sugar derivatives.

### 2.3. Chemical Composition Analysis of T. chebula by Key Ion Diagnostics and Neutral Loss Filtering Using UPLC-Q-TOF/MS

Mass spectrometry analysis of gallic tannin or glucose gallate frequently reveals the neutral loss of multiple galloyl groups and gallic esters, as evidenced by the observation of fragments such as [M–H–152]^−^ and [M–H–170]^−^. These characteristic neutral loss fragments serve as valuable indicators for deducing the molecular structure. Additionally, the primary mass spectrum typically displays high-intensity [M–2H]^2−^ peaks for macromolecular components containing multiple galloyl groups, while derivatives with fewer galloyl groups primarily generate [M–H]^−^ or [M+HCOO]^−^ adduct ions. However, misinterpretation may occur wherein the [M–2H]^2−^ peak is incorrectly identified as [M–H]^−^, leading to the misidentification of the true [M–H]^−^ peak as [2M–H]^−^. To mitigate the potential for such misjudgments, the secondary mass spectrum is generally employed for confirmation. A comparative analysis of the primary and secondary mass spectra reveals that fragments exhibiting higher intensity in the primary mass spectrum often disappear in the secondary mass spectrum, indicating their status as [M–2H]^2−^ characteristic peaks. Concurrently, fragments that exhibit reduced intensity in the secondary mass spectrum can be more reliably assigned as [M–H]^−^. This analytical approach significantly enhances the reliability of structural analysis for the target compound by effectively reducing the risk of misidentification.

By using the UPLC-Q-TOF/MS instrument, (Waters Corp., Manchester, UK) the *T. chebula* extract could be analyzed within 33 min, and the data were processed by KID-NLF. As a result, a total of 164 compounds were rapidly identified (Figure 2 and Figure 3 and Table 1). They could be divided into three groups according to their structural types and MS/MS fragmentation pathways.

#### 2.3.1. Gallic Acid Derivatives

In *T. chebula*, the primary compounds of gallic acid and its derivatives consist of simple gallic acid components and their acyl esters. The main constituents identified are gallic acid (**8**) and methyl gallate (**39**). These compounds are characterized by multiple phenolic hydroxyl and carboxyl groups, which render them susceptible to the loss of molecules such as CO_2_, CO, and H_2_O during cleavage. A relatively high content of gallic acid is present in myrobalan, with two significant ion peaks observed at *m*/*z* 169 and *m*/*z* 125 in the primary mass spectrum. Based on the peak elution time and ionic strength of the compounds, *m*/*z* 169 has been confirmed as the deprotonated molecular ion [M–H]^−^, while *m*/*z* 125 is identified as the main fragment ion resulting from in-source fragmentation, with the molecular formula C_7_H_6_O_5_. Further analysis indicates that the primary secondary fragment is the ion at *m*/*z* 125.0238 [M–H–CO_2_]^−^, formed by the removal of a carboxyl group (CO_2_, Δm = 44). By comparing with the mass spectrometry data of the reference substance, peak **8** has been confirmed as gallic acid. The primary mass spectrum of peak **39** exhibits a prominent abundance at *m*/*z* 183, which is identified as a deprotonated molecular ion [M–H]^−^, corresponding to the molecular formula C_8_H_8_O_5_. In the secondary mass spectrum, in addition to the *m*/*z* 169 and *m*/*z* 125 fragments of gallic acid, characteristic fragments *m*/*z* 168 [M–H–CH_3_]^−^ and *m*/*z* 124.0160 [M–H–CH_3_−CO_2_]^−^ have also been detected. Consequently, peak **39** has been confirmed as methyl gallate.

**Figure 5:**
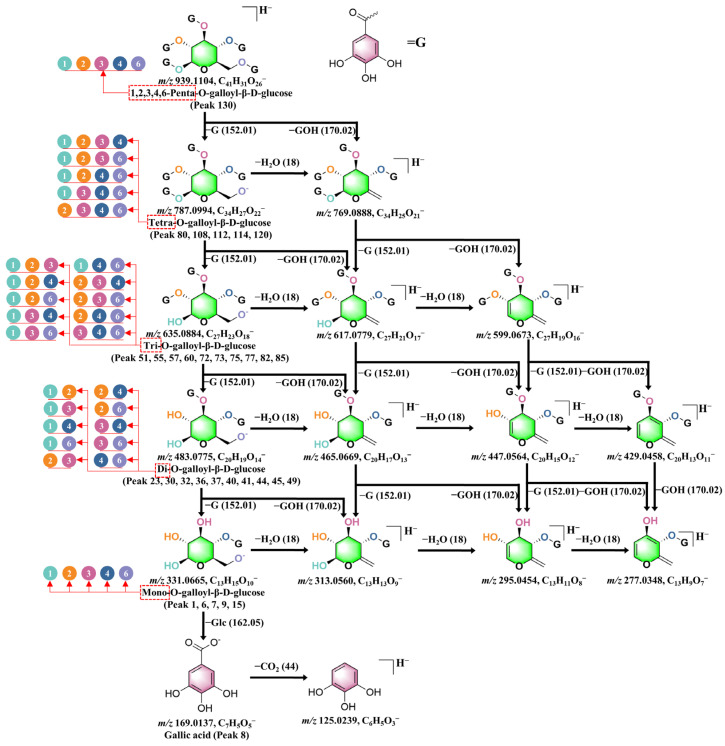
Schematic putative fragmentation pattern of gallotannins. The positions of galloyl groups on the sugar structure are indicated by numbers in circular shapes of different colors. The number of galloyl groups in glycoside is shown by red dashed boxes.

Simple galloyl ester compounds are mainly divided into three categories: gallic acid bound to glucose (Glc), gallic acid bound to quinic acid, and gallic acid bound to shikimic acid. In this study, 38 gallotannin components were identified. These components were classified according to the number of galloyl groups attached to the Glc molecules. The categories include mono-galloyl glucose (**1**, **6**, **7**, **9**, **15**), di-galloyl glucose (**23**, **30**, **32**, **36**, **37**, **40**, **41**, **44**, **45**, **49**), tri-galloyl glucose (**51**, **55**, **57**, **60**, **72**, **73**, **75**, **77**, **82**, **85**), tetra-galloyl glucose (**80**, **108**, **112**, **114**, **120**), and penta-galloyl glucose (**130**). Characteristic neutral losses were observed as galloyl and gallic acid moieties were gradually removed (Figure 5).

The mass spectrum of penta-galloyl glucose (*m*/*z* 939) demonstrated a sequential loss of galloyl groups, resulting in the formation of tetra-galloyl glucose (*m*/*z* 787), tri-galloyl glucose (*m*/*z* 635), di-galloyl glucose (*m*/*z* 483), and mono-galloyl glucose (*m*/*z* 331). Additionally, the primary intermediate ions observed for di-galloyl glucose and mono-galloyl glucose were *m*/*z* 271 and *m*/*z* 211, respectively. These fragment ions were attributed to the continuous loss of -CHOH groups from the glucose moiety, indicating that mono-galloyl glucose undergoes fragmentation to yield [M–H–60]^−^ and [M–H–60–60]^−^.

**Figure 6:**
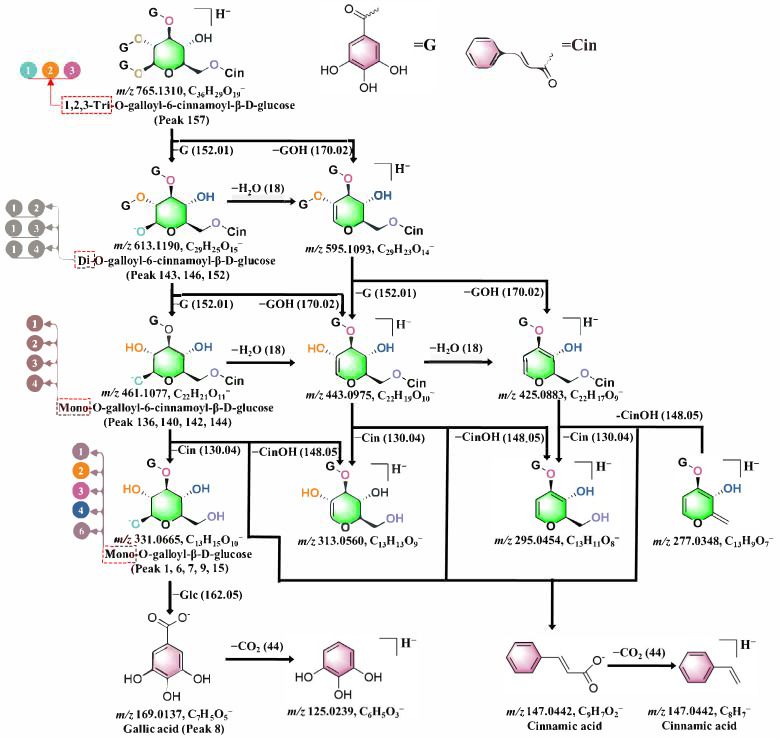
Schematic putative fragmentation pattern of galloyl derivatives of cinnamic acid. The positions of galloyl groups on the sugar structure are indicated by numbers in circular shapes of different colors. The number of galloyl groups in glycoside is shown by red dashed boxes.

Based on the number and structure of galloyl and cinnamoyl groups, a series of isomers can be distinguished, and their peaks can be clearly identified. These compounds are categorized as cinnamoyl-mono-galloyl glucose (**136**, **140**, **142**, **144**), cinnamoyl-di-galloyl glucose (**143**, **146**, **152**), and cinnamoyl-tri-galloyl glucose (**157**). In the mass spectrum, cinnamoyl-tri-galloyl glucose (*m*/*z* 765) exhibits a gradual loss of the gallic acid component, leading to the formation of cinnamoyl-di-galloyl glucose (*m*/*z* 595), cinnamoyl-mono-galloyl glucose (*m*/*z* 425), and cinnamoyl glucose (*m*/*z* 255) (Figure 6). Additionally, characteristic diagnostic ions of *m*/*z* 169, attributed to gallic acid, are observed in the mass spectrometry analysis. Ion fragments of *m*/*z* 125 are generated through the neutral loss of carboxyl groups (CO_2_, Δm = 44), while *m*/*z* 103 is formed by the loss of carboxyl groups (CO_2_, Δm = 44) from cinnamic acid, serving as a distinctive fragment for cinnamic acid. For the position isomers, the calculated lipophilicity parameter (ClogP) was used to estimate the retention time of isomers in the reversed-phase column as the basis for differentiation. Generally, compounds with a larger ClogP value would retain longer. The structures of these isomers were ultimately assigned by combining peak times with calculated ClogP values (Table S1).

**Figure 7:**
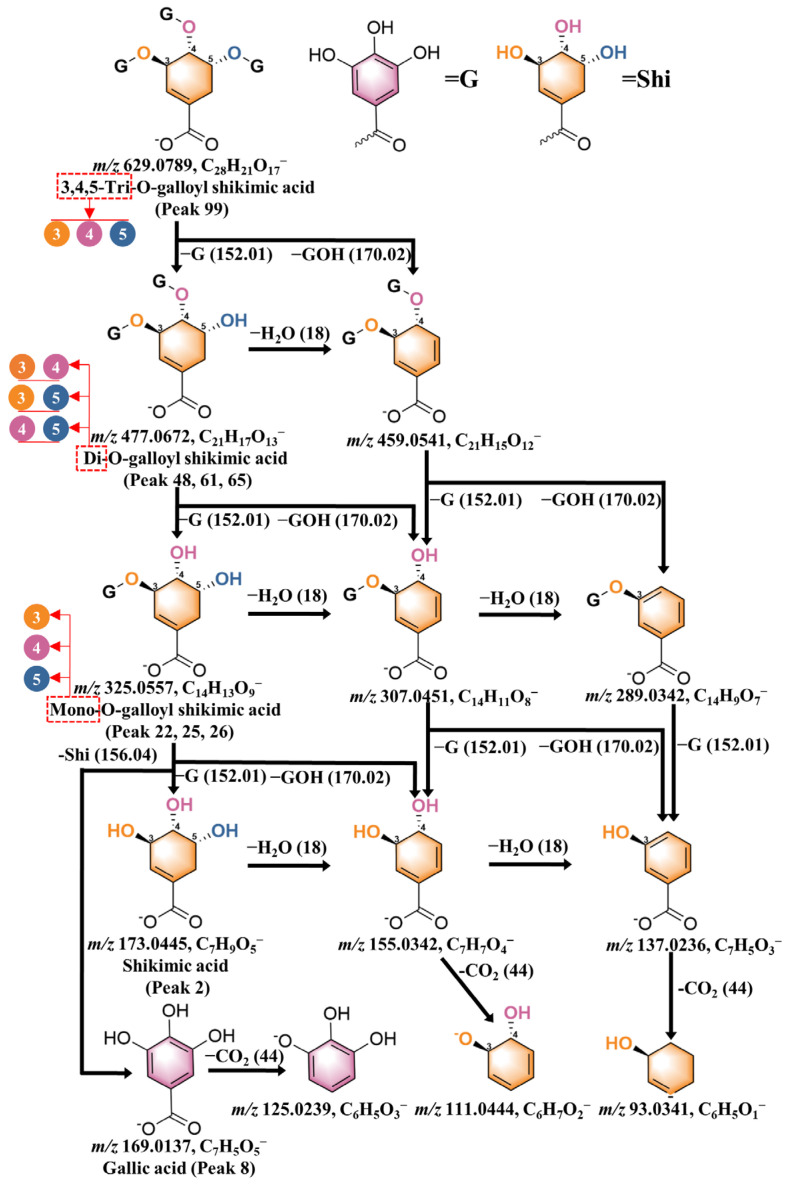
Schematic putative fragmentation pattern of galloyl derivatives of shikimic acid. The positions of galloyl groups on the shikimic acid structure are indicated by numbers in circular shapes of different colors. The number of galloyl groups in shikimic acid is shown by red dashed boxes.

Another type of simple galloyl ester is formed by the combination of gallic acid and shikimic acid. Based on the number of galloyl groups, these compounds can be categorized as mono-galloyl shikimic acid, di-galloyl shikimic acid, and tri-galloyl shikimic acid. In this study, a total of seven related compounds were identified and classified according to the number of galloyl groups connected to shikimic acid, including mono-galloyl shikimic acid (**22**, **25**, **26**), di-galloyl shikimic acid (**48**, **61**, **65**), and tri-galloyl shikimic acid (**99**). Prominent characteristic fragment ions were observed in the mass spectrum through the sequential elimination of galloyl and shikimic acid moieties. Tri-galloyl shikimic acid (*m*/*z* 629) demonstrated continuous mass loss of the galloyl moiety, leading to the formation of di-galloyl shikimic acid (*m*/*z* 477) and mono-galloyl shikimic acid (*m*/*z* 325). A fragment ion of *m*/*z* 169 was detected for all components and identified as [M–H]^−^ of gallic acid, resulting in a fragment of *m*/*z* 125 through the neutral loss of a carboxyl group (CO_2_, Δm = 44). In addition, fragment ions of *m*/*z* 155 and *m*/*z* 137 were attributed to the loss of water molecules (H_2_O, Δm = 18) from shikimic acid. Similarly, further removal of the carboxyl group (CO_2_, Δm = 44) after water loss resulted in fragment ions of *m*/*z* 111 and *m*/*z* 93, which are considered characteristic fragments of shikimic acid. Finally, by analyzing the arrangement of galloyl groups at different positions on shikimic acid, a series of isomers was identified (Figure 7). The assignment of each peak was determined by examining peak times and ClogP values (Table S1).

The final type of simple galloyl ester is formed by the combination of gallic acid and quinic acid. This compound typically produces a characteristic fragment at *m*/*z* 191. While components with two or three galloyl groups combined with quinic acid may exist, none were identified in this study. A total of three isomers of mono-galloyl quinic acid were identified. In the primary mass spectrum, peaks **5**, **11**, and **17** all displayed ion peaks at *m*/*z* 389 and *m*/*z* 343. Notably, the intensity of peak **17** was significantly higher than that of peaks **5** and **11**, approximately five times greater. Additionally, peak **17** presented fragments at *m*/*z* 709 and *m*/*z* 687. In the secondary mass spectrum, the ion peak intensities of *m*/*z* 709, *m*/*z* 687, and *m*/*z* 389 were all reduced. Based on these observations, it was concluded that *m*/*z* 709 corresponds to the [2M+Na–2H]^−^ ion peak, *m*/*z* 687 corresponds to the [2M–H]^−^ ion peak, and *m*/*z* 389 represents the adduct ion [M+HCOO]^−^, with the corresponding molecular formula being C_16_H_14_O_10_. Fragment ion peaks of *m*/*z* 191 [quinic acid–H]^−^ and *m*/*z* 169 [gallic acid–H]^−^ can be generated through the neutral loss of quinic acid or gallic acid. By incorporating relevant literature, the order of the peaks was further clarified, leading to the identification of peak **5** as 3-galloyl quinic acid, peak **11** as 5-galloyl quinic acid, and peak **17** as 4-galloyl quinic acid.

#### 2.3.2. Ellagitannins

Ellagitannins are a significant class of polyphenolic compounds containing one or more hexahydroxydiphenoyl (HHDP) groups or their oxidized forms, such as dehydrohexahydroxydiphenoyl (DHHDP) and chebuloyl (Che). Upon hydrolysis, they yield stable ellagic acid. In negative ion mode, ellagic acid tannins exhibit characteristic fragment ion losses, including HHDP (302 Da), DHHDP (318 Da), and chebuloyl (320 Da) (Figure 4).

##### Ellagic Acid and Its Simple Derivatives

The simple derivatives of ellagic acid primarily consist of ellagic acid conjugated with rhamnose to form glycosides at the 1-position, with additional hydroxyl groups on the rhamnose linked to multiple galloyl groups. MS analysis reveals that peaks **134**, **137**, and **138** display identical ions at *m*/*z* 375 and *m*/*z* 751 in the primary MS. The disappearance of *m*/*z* 375 ions in the secondary MS suggests that *m*/*z* 375 corresponds to the [M–2H]^2^^−^ ion peak. The *m*/*z* 751 ion is identified as the [M–H]^−^ ion peak, corresponding to the molecular formula C_34_H_24_O_20_. In the secondary mass spectrum, fragment ions were observed at *m*/*z* 599 [M–H–152]^−^, *m*/*z* 581 [M–H–170]^−^, *m*/*z* 449 [M–H–302]^−^, *m*/*z* 411 [M–H–170–170]^−^, and *m*/*z* 301 [M–H–152–152–146]^−^/[ellagic acid–H]^−^, generated by the sequential loss of galloyl, gallic, and ellagic acid groups. The polarity was assessed using ClogP values (Table S1), leading to the identification of peak **134** as 4*-O-*(2″,3″-di-*O*-galloyl-α-L-rhamnosyl) ellagic acid, peak **137** as 4-*O*-(2″,4″-di-*O*-galloyl-α-L-rhamnosyl) ellagic acid and peak **138** as 4-*O*-(3″,4″-di-*O*-galloyl-α-rhamnopyranosyl) ellagic acid. Similar cleavage patterns were observed for peaks **102** and **132**, where peak **102** was identified as Eschweilenol C and peak **132** as 4-*O*-(4″-*O*-galloyl-α-rhamnopyranosyl) ellagic acid. Additionally, ellagic acid can directly conjugate with gallic acid. In the primary mass spectrum of peak **29**, ion peaks were observed at *m*/*z* 469 and *m*/*z* 939. The intensity of the *m*/*z* 939 ion peak diminished in the secondary mass spectrum, indicating that *m*/*z* 939 corresponds to the [2M–H]^−^ ion peak, while *m*/*z* 469 corresponds to the [M–H]^−^ ion peak, with the molecular formula C_21_H_10_O_13_. Fragment ion peaks at *m*/*z* 425 [M–H–CO_2_]^−^, *m*/*z* 407 [M–H–CO_2_−H_2_O]^−^, and *m*/*z* 299 [M–H–gallic acid]^−^ were generated by the loss of CO_2_ and H_2_O. Peak **29** was identified as Valoneic acid dilactone. *Terminalia chebula* contains a significant amount of free ellagic acid. Numerous studies have focused on the quantification of ellagic acid using HPLC/UPLC. Peak **88** was identified as ellagic acid through comparison with reference standards.

##### Simple Tannins Containing a Single HHDP Group

Simple tannins containing a single HHDP group are primarily composed of compounds with one HHDP group and may include one or more attached galloyl groups. Based on the number of galloyl groups, these compounds can be classified into three types: mono-galloyl-HHDP glucose (**10**, **18**, **38**, **50**, **71**), di-galloyl-HHDP glucose (**59**, **68**, **76**, **110**, **117**), and tri-galloyl-HHDP glucose (**81**, **91**, **97**, **104**, **107**, **135**). In mass spectrometry, these compounds exhibit a characteristic cleavage pattern, initially losing the HHDP group, followed by sequential losses of galloyl groups. The mass spectrum shows a characteristic neutral loss of 302 Da, indicative of HHDP group cleavage. For instance, tri-galloyl-HHDP glucose (*m*/*z* 937) first loses the HHDP group to generate tri-galloyl glucose (*m*/*z* 635), which then fragments following the cleavage pathway typical for tri-galloyl glucose. Similarly, mono-galloyl-HHDP glucose and di-galloyl-HHDP glucose exhibit comparable fragmentation patterns. Additionally, this class of compounds also produces a characteristic fragment at *m*/*z* 275, primarily generated by the elimination of the HHDP group, followed by the loss of a galloyl group and subsequently glucose.

##### Chebulic Acid and Its Simple Derivatives

The primary chebulic acids found in *Terminalia chebula* are neochebulic acid (**3**), chebulic acid (**4**), and isochebulic acid (**12**). These compounds are rich in phenolic hydroxyl and carboxyl groups, making them prone to losing CO_2_, H_2_O, and other groups during mass spectrometry fragmentation. In the primary mass spectrum, ion peaks at *m*/*z* 711, *m*/*z* 355, and *m*/*z* 337 correspond to peaks **3**, **4**, and **12**, respectively. These ion peaks are also observed in the secondary mass spectrum. The *m*/*z* 711 peak is presumed to be the [2M–H]^−^ ion, *m*/*z* 355 as the [M–H]^−^ ion, and *m*/*z* 337 as a fragment with the molecular formula C_14_H_12_O_11_. During pyrolysis, the sequential loss of H_2_O and CO_2_ generates fragment ion peaks at *m*/*z* 293 [M–H–H_2_O–CO_2_]^−^, *m*/*z* 249 [M–H–H_2_O–2CO_2_]^−^, and *m*/*z* 205 [M–H–H_2_O–3CO_2_]^−^. The polarity was assessed using ClogP values (Table S1), and peak order was established by comparison with reference standards. Consequently, peak **3** was identified as isochebulidic acid, peak **4** as chebulidic acid, and peak **12** as neochebulidic acid. In the primary mass spectrum, peaks **21** and **28** exhibit ion peaks at *m*/*z* 369 and *m*/*z* 739, respectively. In the secondary mass spectrum, the intensity of the *m*/*z* 739 ion peak diminishes, suggesting that *m*/*z* 739 corresponds to a [2M–H]^−^ ion, while *m*/*z* 369 corresponds to a [M–H]^−^ ion, with the molecular formula C_15_H_14_O_11_. During pyrolysis, these compounds produce fragment ion peaks at *m*/*z* 351 [M–H–H_2_O]^−^, *m*/*z* 325 [M–H–CO_2_]^−^, and *m*/*z* 307 [M–H–H_2_O−CO_2_]^−^ through the sequential loss of H_2_O and CO_2_. Peak **21** was identified as 7′-*O*-methylchebulate and peak **28** as 6′-*O*-methylchebulate.

##### Simple Tannins Containing a Single Chebuloyl or Neoche Group

In this study, the primary types of simple tannin-like components within the chebuloyl, neoche, and methylneoche groups were identified through mass spectrometry. These components primarily consist of a chebuloyl (320 Da), neoche (338 Da), or methylneoche (352 Da) group and their related derivatives, potentially containing multiple galloyl groups. The molecular weight difference between the chebuloyl and neoche groups is equivalent to one H_2_O molecule (18 Da). The key distinction between these groups lies in the attachment: the chebuloyl group is linked to two glycohydroxyl groups, while the neoche group is linked to only one. Based on the number of galloyl groups, these compounds can be further categorized as mono galloyl-neoche glucose (**24**, **27**, **33**, **35**, **42**, **52**, **53**, **56**, **62**, **63**, **66**), bisgalloyl-chebuloyl glucose (**78**, **92**, **98**), and trigaloyl-chebuloyl/neoche glucose (**83**, **94**, **100**, **106**, **111**, **122**, **124**, **125**, **126**, **127**, **128**, **129**). These compounds exhibited neutral losses of 320 Da, 338 Da, or 352 Da in the mass spectrum. This indicates that these compounds are prone to losing chebuloyl, neoche, or methylneoche groups during mass spectrometric analysis. Specifically, the fragmentation of trigaloyl-chebuloyl/neoche glucose results in the loss of the chebuloyl/neoche group, forming trigaloyl glucose (*m*/*z* 635), which then undergoes further cleavage following the pattern typical of trigaloyl glucose. Likewise, monogalloyl-chebuloyl/neoche glucose displayed a comparable fragmentation pattern.

##### Tannins Containing HHDP, Chebuloyl, Neoche, and Other Groups

*Terminalia chebula* contains various tannins, which typically include groups such as HHDP (302 Da), chebuloyl (320 Da), neoche (338 Da), THDP (292 Da), DHHDP (318 Da), flavogallonyl (452 Da), and Gallagyl (602 Da). The combinations of these groups are complex and varied, potentially including HHDP along with other groups such as chebuloyl, neoche, THDP, DHHDP, Gallagyl, or flavogallonyl. Different components can be distinguished and identified based on the characteristic neutral loss of these groups and their related fragments.

**Figure 8:**
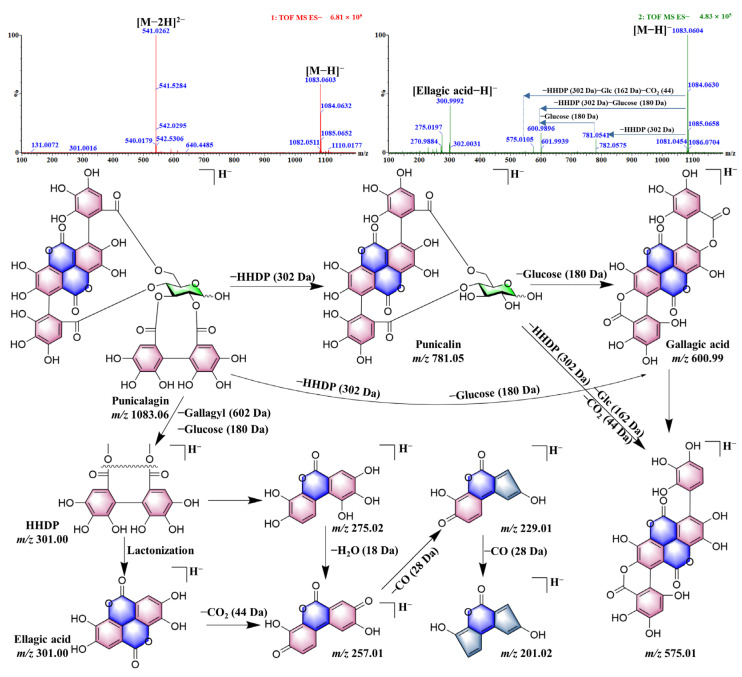
The putative fragmentation pathway of Punicalagin.

In mass spectrometry, peaks **19**, **20**, **31**, **43**, and **84** display identical ions at *m*/*z* 541 and 1083 in the primary mass spectrum. The *m*/*z* 541 ion disappears in the secondary mass spectrum and is identified as [M–2H]^2^^−^, while *m*/*z* 1083 is identified as the [M–H]^−^ ion with the molecular formula C_48_H_28_O_30_. The secondary mass spectrum reveals that these compounds generate major fragment ions at *m*/*z* 601 [M–H–HHDP–glucose]^−^ and *m*/*z* 300 [ellagic acid–H]^−^, indicating significant neutral losses of HHDP and glucose. Peaks **19** and **20** also exhibit characteristic fragments at *m*/*z* 451 [Flavogallonic acid–H–H_2_O]^−^. Peak **19** is identified as Punicacortein C and peak 20 as Punicacortein D based on the literature comparison. Peaks **31** and **43** are characterized by *m*/*z* 781 [M–H–HHDP]^−^ fragments. The retention time and fragmentation pathway of peaks **31** and **43** were confirmed to correspond to Punicalagin-α and Punicalagin-β; the fragmentation pathway of Punicalagin is showed in Figure 8. Peak **84** is also characterized by *m*/*z* 449 [M–H–HHDP–glucose–galloyl]^−^, identified as *T. chebula* based on its retention time. The ions at *m*/*z* 542 and 1085 are observed in the primary mass spectrum, while *m*/*z* 542 disappears in the secondary spectrum. These are identified as [M–2H]^2^^−^ and *m*/*z* 1085 as [M–H]^−^ with the molecular formula C_48_H_30_O_30_. Through the loss of HHDP and galloyl groups, the fragment ions *m*/*z* 783 [M–H–HHDP]^−^, *m*/*z* 631 [M–H–HHDP–galloyl]^−^, and characteristic fragments at *m*/*z* 451 [Flavogallonic acid–H–H_2_O]^−^ are generated. Based on ClogP values (Table S1) and the literature, peak **34** was identified as Rhoipteleanin G and peak **46** as Terflavin A. The ions at *m*/*z* 494 and 989 are observed in the primary mass spectrum, while *m*/*z* 494 disappears in the secondary spectrum. These ions are identified as [M–2H]^2^^−^ and *m*/*z* 989 as [M–H]^−^, with the molecular formula C_41_H_34_O_29_. The compound first loses a neoche group, forming the *m*/*z* 651 [M–H–neoche]^−^ fragment, and then loses a gallic acid to generate the *m*/*z* 481 [M–H–neoche–gallic acid]^−^ fragment. The characteristic fragment at *m*/*z* 337 [neochebulic acid–H–H_2_O]^−^ is generated, and peak **58** is identified as Carpinusnin.

Peaks **64**, **67**, **70**, **74**, **79**, **87**, **101**, **109**, **118**, and **121** display ions at *m*/*z* 485 and 971 in the primary mass spectrum. The *m*/*z* 485 ion disappears in the secondary mass spectrum, while *m*/*z* 971 is identified as an [M–H]^−^ ion with the molecular formula C_41_H_32_O_28_. These compounds are detected in the secondary mass spectrum through the loss of groups such as gallic acid, HHDP, and neoche. The fragment ions observed include *m*/*z* 801 [M-H-170]^−^, *m*/*z* 669 [M–H–302]^−^, *m*/*z* 633 [M–H–338]^−^, *m*/*z* 499 [M–H–170–302]^−^, *m*/*z* 463 [M–H–338–302]^−^, *m*/*z* 337 [neochebulic acid–H–H_2_O]^−^, and *m*/*z* 301 [ellagic acid–H]^−^. Based on the chemical structure of components in *Terminalia chebula*, HHDP is likely attached to the 3,6, 4,6, or 2,3 hydroxyl groups of glucose, while neoche groups generally do not attach to the 1-hydroxyl group of glucose. Using ClogP values (Table S1) to assess polarity, the following isomer structures were inferred and confirmed: Peak **64** corresponds to 4-galloyl-6-neoche-2,3-HHDP-glucose and peak **67** to 1-galloyl-2-neoche-4,6-HHDP-glucose. Peak **70** is identified as 1-galloyl-3-neoche-4,6-HHDP-glucose and peak **74** as 1-galloyl-2-neoche-3,6-HHDP-glucose. Peak **79** corresponds to 1-galloyl-4-neoche-3,6-HHDP-glucose and peak **87** to 1-galloyl-4-neoche-2,3-HHDP-glucose. Peak **101** is identified as 2-galloyl-3-neoche-4,6-HHDP-glucose and peak **109** as 1-galloyl-6-neoche-2,3-HHDP-glucose. Peak **118** corresponds to 2-galloyl-4-neoche-3,6-HHDP-glucose and peak **121** to 6-galloyl-4-neoche-2,3-HHDP-glucose.

**Figure 9:**
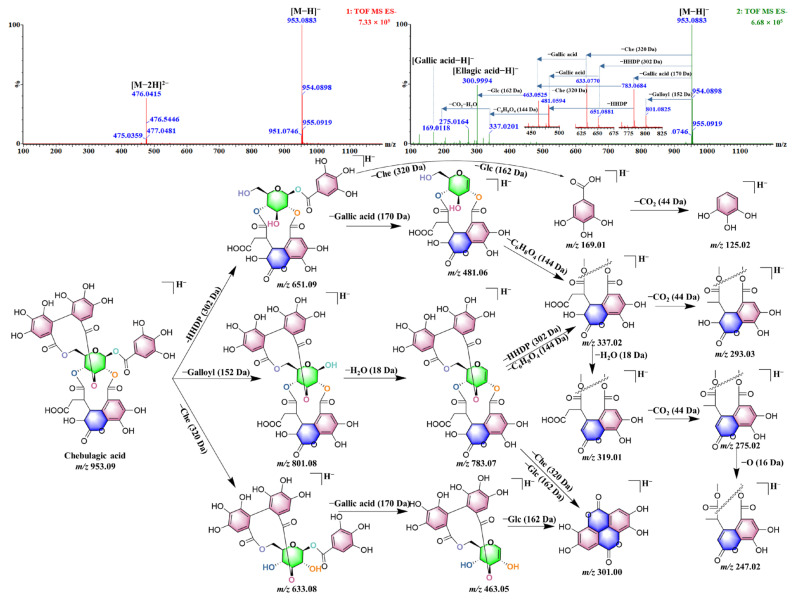
The putative fragmentation pathway of chebulagic acid.

In the primary mass spectrum, the ion signal at *m*/*z* 953 was observed in peaks **86**, **90**, **95**, **105**, **119**, and **131**, while the ion at *m*/*z* 476 was detected in peaks **86**, **90**, **95**, **105**, **119**, and **123**, but not at peak **131**. In the secondary mass spectrum, the signal at *m*/*z* 476 disappeared. Thus, *m*/*z* 476 was inferred to be the [M–2H]^2^^−^ ion peak, and *m*/*z* 953 was identified as the [M–H]^−^ ion peak. The molecular formula was determined to be C_41_H_30_O_27_. Further analysis of the secondary mass spectrum indicates that the fragment patterns of peaks **86**, **90**, **95**, **105**, **119**, and **123** are very similar. The main fragment ions observed include *m*/*z* 783 [M–H–170]^−^, *m*/*z* 651 [M–H–302]^−^, *m*/*z* 633 [M–H–320]^−^, *m*/*z* 481 [M–H–302–170]^−^, *m*/*z* 463 [M–H–320–170]^−^, *m*/*z* 337 [chebuloyl–H]^−^, *m*/*z* 331 [M–H–320–302]^−^, *m*/*z* 319 [M–H–302–170–162]^−^, and *m*/*z* 301 [ellagic acid–H]^−^. These fragment ions primarily result from the loss of groups such as gallic acid, chebuloyl, HHDP, and glucose. Based on the fragment ion information, six isomers were identified. Peak **105** was identified as chebulagic acid by comparing its retention time and intensity with those of a reference standard; the fragmentation pathway of chebulagic acid is showed in Figure 9. By assessing the polarity of each peak using ClogP values (Table S1), the following structures were assigned: Peak **86** corresponds to 1-*O*-galloyl-3,4-chebuloyl-2,6-HHDP-D-glucose, and peak **90** corresponds to 1-*O*-galloyl-3,6-chebuloyl-2,4-HHDP-D-glucose. Peak **95** was assigned as 1-*O*-galloyl-4,6-chebuloyl-3,3-HHDP-D-glucose and peak **119** as 1-*O*-galloyl-2,6-chebuloyl-3,4-HHDP-D-glucose. Peak **123** was assigned as 1-*O*-galloyl-2,3-chebuloyl-4,6-HHDP-D-glucose. The secondary fragments of peak **131** mainly include *m*/*z* 935.0799 [M–H–H_2_O]^−^, *m*/*z* 917.0695 [M–H–2H_2_O]^−^, *m*/*z* 635.0896 [M–H–DHHDP]^−^, *m*/*z* 617.0781 [M–H–H_2_O– DHHDP]^−^, *m*/*z* 465.0671 [M–H–DHHDP–gallic acid]^−^, and *m*/*z* 316.9932 [DHHDP–H]^−^. The loss of DHHDP (318 Da) is the main neutral loss characteristic of the compound, suggesting that peak **131** is terchebin.

Peaks **113** and **115** display identical ions at *m*/*z* 492 and 985 in the primary mass spectrum. The *m*/*z* 492 ion disappears in the secondary mass spectrum, indicating that *m*/*z* 492 corresponds to the [M–2H]^2^^−^ ion, while *m*/*z* 985 is the [M–H]^−^ ion, with the molecular formula determined as C_42_H_34_O_28_. In the secondary mass spectrum, fragment ions were observed at *m*/*z* 815 [M–H–170]^−^, *m*/*z* 683 [M–H–302]^−^, *m*/*z* 633 [M–H–352]^−^, *m*/*z* 513 [M–H–302–170]^−^, *m*/*z* 463 [M–H–352–170]^−^, *m*/*z* 351 [6′-*O*-methyl neochebulic acid-H_2_O-H]^−^, and *m*/*z* 301 [ellagic acid–H]^−^. These ions are generated through the sequential loss of gallic acid, HHDP, and 6′-*O*-methyl neochebuloyl. Based on the location of the galloyl group, two isomers were deduced, and their polarity was determined using ClogP values (Table S1). Peak **113** corresponds to 1-*O*-galloyl-3,6-HHDP-4-6′-methyl neochebuloyl-glucose, while peak **115** corresponds to 2-*O*-galloyl-3,6-HHDP-4-6′-methyl neochebuloyl-glucose.

Peaks **89**, **93**, **96**, **103**, **116**, and **133** display identical ions at *m*/*z* 462 and 925 in the primary mass spectrum. The ion at *m*/*z* 462 disappears in the secondary mass spectrum, indicating that it corresponds to the [M–2H]^2^^−^ ion, while *m*/*z* 925 is assigned to the [M–H]^−^ ion. The molecular formula is determined to be C_40_H_30_O_26_. In the secondary mass spectrum, fragment ions were observed at *m*/*z* 773 [M–H–152]^−^, *m*/*z* 633 [M–H–292]^−^, *m*/*z* 481 [M–H–292–152]^−^, and *m*/*z* 465 [M–H–292–170]^−^, resulting from the sequential loss of galloyl, THDP, and other groups. Based on the positions of the galloyl and THDP groups, six isomers were deduced, and their polarity was determined using ClogP values (Table S1). Peak **89** was identified as 1-*O*-galloyl-3,4-THDP-2,6-HHDP-D-glucose. Peak **93** was identified as 1-*O*-galloyl-2,4-THDP-6,6-HHDP-D-glucose (Phyllanthusiin C) and peak **96** as 1-*O*-galloyl-3,6-THDP-2,4-HHDP-D-glucose. Peak **103** was identified as 1-*O*-galloyl-4,6-HHDP-2,3-HHDP-D-glucose, while peak **116** was identified as 1-*O*-galloyl-2,3-HHDP-4,6-HHDP-D-glucose. Peak **133** was identified as 1-*O*-galloyl-2,6-HHDP-3,4-HHDP-D-glucose.

#### 2.3.3. Terpenoids

Terpenoids generate abundant deprotonated molecular ions [M–H]^−^ in primary mass spectrometry, with some also forming [2M–H]^−^ ions. These characteristics facilitate the identification of excimer ions and the determination of their molecular formulas. The saponins in *Terminalia chebula* are typically linked to glucose (Glc), galloyl, and glucoheptonic acid through various substitution patterns. Aglycone fragments are relatively stable, primarily losing sugar and galloyl fragments (152, 162, 208 Da) under normal voltages. By analyzing the fragment ions of saponins, aglycones can be classified into 503 Da and 487 Da groups, each with multiple core configurations.

Peaks **139**, **149**, and **153** exhibit a high-intensity ion signal at *m*/*z* 711 in the primary mass spectrum, identified as the [M–H]^−^ ion peak corresponding to the molecular formula C_37_H_60_O_13_. In the secondary mass spectrum, the fragment at *m*/*z* 503 [M–H–208]^−^ displayed a high-intensity signal, suggesting that it resulted from the loss of glucoheptonic acid. Based on the saponin characteristics, the fragment at *m*/*z* 503 is attributed to the loss of glucoheptonic acid. Peak **139** was identified as Arjungenin-24-*O*-glucoheptonic acid, and peak 149 as Madecassic acid-24-*O*-glucoheptonic acid. Peak **153** was identified as Terminolic acid-24-*O*-glucoheptonic acid. Peaks **141**, **145**, and **147** exhibit strong ion signals at *m*/*z* 817 in the primary mass spectrum. Combined with secondary mass spectrometry, *m*/*z* 817 is identified as the [M–H]^−^ ion peak, corresponding to the molecular formula C_43_H_62_O_15_. The secondary mass spectrum reveals intense fragment ions at *m*/*z* 655 [M–H–162]^−^ and *m*/*z* 503 [M–H–162–152]^−^, likely resulting from the loss of glucose and galloyl groups, as indicated by the characteristic fragments at *m*/*z* 503. **Peak 141** was identified as Quercotriterpenoside I (Arjungenin-24-galloyl-28-glucose), **peak 145** as Madecassic acid-24-galloyl-28-glucose, and peak **147** as Terminolic acid-24-galloyl-28-glucose. Peaks **158**, **159**, and **161** exhibit similar ion signals at *m*/*z* 503, 549, and 1007 in the primary mass spectrum. The *m*/*z* 1007 peak is identified as the [2M–H]^−^ ion, while the *m*/*z* 549 peak corresponds to the [M+HCOO]^−^ ion, with a molecular formula of C_30_H_48_O_6_. Peak **158** was identified as Arjungenin, peak **159** as Madecassic acid, and peak **161** as Terminolic acid.

Peaks **148**, **151**, and **155** appear as [M–H]^−^ ion peaks at *m*/*z* 801, corresponding to the molecular formula C_43_H_62_O_14_. The intense fragments at *m*/*z* 639 [M–H–162]^−^ and *m*/*z* 487 [M–H–162–152]^−^ in the secondary mass spectrum suggest that the characteristic fragments at *m*/*z* 487 result from the loss of glucose and gallic acid groups. Peak **148** was identified as Rotundic acid-24-galloyl-28-glucose and peak **151** as Asiatic acid-24-galloyl-28-glucose. Peak **155** was identified as Arjunolic acid-24-galloyl-28-glucose. Peaks **150**, **154**, and **156** all appear as [M–H]^−^ ion peaks at *m*/*z* 695, with the molecular formula identified as C_37_H_60_O_12_. In the secondary mass spectrum, an intense fragment at *m*/*z* 487 [M–H–208]^−^ was observed, which was attributed to the loss of glucoheptonic acid. Peak **150** was identified as Rotundic acid-24-*O*-glucoheptonic acid. Peak **154** was identified as Asiatic acid-24-*O*-glucoheptonic acid and peak 156 as Arjunolic acid-24-*O*-glucoheptonic acid. Peak **160** exhibits a strong ion signal at *m*/*z* 639, corresponding to the molecular formula C_37_H_52_O_9_. In the secondary mass spectrum, a fragment at *m*/*z* 487 [M–H–152]^−^ was detected, attributed to the loss of a galloyl group. Peak **160** was identified as 23-galloyl-arjunolic acid. Peaks **162**, **163**, and **164** show ion peaks at *m*/*z* 487 and 975 in the primary mass spectrum. The *m*/*z* 975 peak is identified as the [2M–H]^−^ ion, while *m*/*z* 487 corresponds to the [M–H]^−^ ion, with a molecular formula of C_30_H_48_O_5_. Peak **162** was identified as Rotundic acid. Peak **163** was identified as Asiatic acid and peak **164** as Arjunolic acid.

#### 2.3.4. Other Components

Besides the previously mentioned ingredients, four additional compounds (**2**, **16**, **47**, **54**, **69**) have been identified in *T. chebula*. Details regarding their chemical compositions are provided in the accompanying table.

### 2.4. The Applicability of the KID-NLF Strategy

The KID-NLF strategy has established identification methods for various medicinal plants by summarizing the research of Moilanen et al. [60] and examining the mass spectrometry rules in *T. chebula*. Employing this strategy allows for the identification of gallic acid derivatives, ellagitannins, and triterpenoids by precisely recognizing quasi-molecular ion peaks and their related secondary characteristic fragments. In addition to *T. chebula*, plants like *Phyllanthus emblica* [61] and *Punica granatum* [62] (rich in tannins) as well as *Panax ginseng*, *P. quinquefolium*, and *P. notoginseng* (containing triterpenoids) [63] also adhere to this identification rule. Further research has shown that iridoid glycosides, phenolic acids, and flavonoids are compatible with this strategy. For instance, iridoid glycosides dipsanosides A and B demonstrate specific mass spectrometry features, such as *m*/*z* 1519.519 [M+HCOO]^−^, 1473.514 [M–H]^−^, 759.252 [M+2HCOO]^2−^, and 736.249 [M–2H]^2−^ as adduct ions. Similarly, the phenolic acids isochlorogenic acid A, B, and C display peaks at *m*/*z* 1029.229 [2M–H]^−^, 537.100 [M+Na–2H]^−^, and 515.118 [M–H]^−^, and the flavonoids hyperoside and isoquercitrin show ions at *m*/*z* 949.164 [2M+Na–2H]^−^, 927.182 [2M–H]^−^, and 463.089 [M–H]^−^ [64,65]. Notably, alkaloids generate primarily [M+H]^+^ type quasi-molecular ions and lack distinctive adduct patterns, making them incompatible with the KID-NLF strategy’s rule system for mass spectrometry. This finding indicates the need for methodological validation based on component type when applying this strategy. Consequently, it is advised to undertake methodological validation according to component type when implementing this strategy and to formulate a specific identification strategy for alkaloids.

## 3. Experimental

### 3.1. Chemicals, Reagents, and Plant Materials

Deionized water was prepared using a Millipore Q purification system (Rephile, Shanghai, China). HPLC-grade acetonitrile, methanol, and formic acid were obtained from Sigma-Aldrich (Milwaukee, WI, USA). The crude medicinal materials derived from dried, pitted mature fruits of *T. chebula* were procured from Chengdu in 2023. The author identified these materials, and the specimens were deposited in the laboratory of the author. Reference products including shikimic acid (**2**), gallic acid (**8**), punicalagin α (**31**), punicalagin β (**43**), corilagin (**71**), ellagic acid (**88**), chebulagic acid (**105**), chebulinic acid (**127**), and 1,2,3,4,6-penta-*O*-galloyl-β-D-glucose (**130**) were purchased from Yuanye (Shanghai, China), with purity exceeding 98% as determined by HPLC analysis.

### 3.2. Sample Preparation

Approximately 1 g of pitted and dried fruit powder of *T. chebula* powder, sieved through a No. 3 mesh, was accurately weighed and transferred into a stoppered conical flask. Subsequently, 50 mL of 70% (*v*/*v*) methanol was added with precision. The sealed flask was weighed and then subjected to ultrasonic treatment at 250 W and 40 kHz for 30 min. After being allowed to cool, the flask was reweighed and any weight loss compensated with 70% methanol. The supernatant was centrifuged at 13,000 rpm for 15 min and filtered through a 0.22 μm microporous filter membrane.

### 3.3. UHPLC Analysis

UHPLC analysis was conducted using a Shimadzu LC-40 system (Shimadzu Corporation, Kyoto, Japan), which was equipped with an SPD-M40 detector. A Shim-pack GIST-C18 column (2.1 × 100 mm, 2 μm) was utilized. The mobile phase consisted of 0.2% phosphoric acid aqueous solution (A) and acetonitrile (B). A gradient elution profile was employed as follows: 0–3 min, 3–6% B; 3–4 min, 6–6% B; 4–5 min, 6–7% B; 5–6 min, 7–7% B; 6–7 min, 7–15% B; 7–10 min, 15–15% B; 10–11 min, 15–21% B; 11–23 min, 21–21% B; 23–26 min, 21–33% B; 26–28 min, 33–35% B; 28–40 min, 35–35%. Detection wavelengths were monitored at 254 nm. The flow rate was set at 0.4 mL/min, injection volume at 2 μL, and column temperature at 30 °C.

### 3.4. UPLC-Q-TOF/MS Analysis

UPLC analysis was carried out using a 100 mm × 2.1 mm, 1.7 μm Waters Acquity UPLCR BEH C18 column (Waters Corporation, Milford, MA, USA). The mobile phase comprised acetonitrile (A) and water (B), each containing 0.1% formic acid. The linear gradient program was set as follows: from 2% to 4% B over 0–2 min; from 4% to 5% B over 2–4 min; from 5% to 6% B over 4–11 min; from 6% to 9% B over 11–17 min; from 9% to 13% B over 17–21 min; maintained at 13% B over 21–27 min; from 13% to 60% B over 27–32 min; from 60% to 99% B over 32–33 min; maintained at 99% B over 33–38 min; from 99% to 1% B over 38–40 min. The flow rate was set at 0.4 mL/min, injection volume at 1 μL, and column temperature at 40 °C.

The Waters SYNAPT G2HDMS system with an ion source was employed for electrospray ionization (ESI). Scanning was conducted in negative (ESI^−^) ion mode, using nitrogen as the atomization and conical gas. The source temperature was set at 100 °C, and the cone gas flow rate was maintained at 40 L/h. The desolvation temperature was held at 350 °C, and the gas flow rate was 800 L/h. Further MS settings included a sampling cone voltage of 40 V, extraction cone voltage of 4 V, capillary voltage of 2.5 kV, scan time of 0.3 s, inter scan time of 0.02 s, and a mass-to-charge ratio (*m*/*z*) ranging from 100 to 1200. Leucine-enkephalin (200 pg/mL) flowing at 10 μL/min was employed to calibrate the mass number *m*/*z* 554.2615. Data processing was conducted using MassLynx V4.2 and UNIFI software (Version 1.9) from Waters Corporation, Milford, MA, USA.

## 4. Conclusions

In conclusion, a post-acquisition LC-MS data processing strategy, key ion diagnostics–neutral loss filtering (KID-NLF), can effectively identify the structure of the natural products responsible for the herbal extract. In this study, a total of 164 compounds were identified by UPLC-Q-TOF/MS technique and KID-NLF strategy screening in 33 min running time, 47 of which were reported for the first time. This study provides a powerful strategy for rapid profiling of chemical constituents of herbal medicines.

## Figures and Tables

**Table 1:** Characterization of chemical constituents in *T. chebula* by UPLC-Q-TOF/MS. (^a^ Isolated from *T. chebula* previously; ^b^ not isolated from *T. chebula* previously; ^c^ a newly discovered ingredient. ^♠^ Gallic acid derivatives; ^♣^ ellagitannins; ^♥^ triterpenoids; ^♦^ others. * Identified by reference standards; ^#^ MS/MS self-built library matching. In MS/MS fragment ions, bold characters with horizontal lines are KID, and bold characters only are NLF.).

No.	Identification	Formula	t_R_ (min)	Experimental (*m*/*z*)	Adducts	Error (ppm)	MS/MS Fragment Ions (*m*/*z*)
1 ^b,#,♠^	3-*O*-galloyl-glucose [17]	C_13_H_16_O_10_	0.71	331.0662	[M–H]^−^	−0.9	**663.1391**, 271.0435, 211.0234, **169.0134**, 125.0235
2 ^a,^*^,♦^	Shikimic acid [18]	C_7_H_10_O_5_	0.75	173.0445	[M–H]^−^	−2.9	155.0342, 137.0235, 93.0341
3 ^a,#,♣^	Neochebulic acid [19]	C_14_H_12_O_11_	0.76	355.0298	[M–H]^−^	−0.8	**711.0679**, 337.0191, 293.0295, 249.0401, 205.0497^−^
4 ^a,#,♣^	Chebulic acid [20]	C_14_H_12_O_11_	0.93	355.0303	[M–H]^−^	0.6	**711.0682**, 337.0194, 293.0297, 249.0402, 205.0501^−^
5 ^b,#,♠^	3-galloylquinic acid [21]	C_14_H_16_O_10_	1.05	343.0661	[M–H]^−^	−1.2	**389.0355**, 191.0572, **169.0141**, 125.0240
6 ^a,#,♠^	1-*O*-galloyl-glucose [22]	C_13_H_16_O_10_	1.17	331.0665	[M–H]^−^	0.0	**663.1403**, 271.0457^−^, 211.0239, **169.0140**, 125.0238
7 ^a,#,♠^	6-*O*-galloyl-glucose [20]	C_13_H_16_O_10_	1.38	331.0666	[M–H]^−^	0.3	**663.1408**, 271.0455, 211.0244, **169.0135**, 125.0238
8 ^a,^*^,♠^	Gallic acid [22]	C_7_H_6_O_5_	1.53	169.0139	[M–H]^−^	1.2	125.0238
9 ^a,#,♠^	2-*O*-galloyl-glucose [23]	C_13_H_16_O_10_	1.55	331.0665	[M–H]^−^	0.0	**663.1407**, 271.0455, 211.0244, **169.0134**, 125.0236
10 ^a,#,♣^	Gemin D [20]	C_27_H_22_O_18_	1.59	633.0726	[M–H]^−^	−0.3	**463.0544**, **300.9984**, 275.0194, **169.0135**, 125.0234
11 ^b,#,♠^	5-galloylquinic acid [21]	C_14_H_16_O_10_	1.72	343.0662	[M–H]^−^	−0.9	**389.0356**, 191.0542, **169.0138**, 125.0236
12 ^a,#,♣^	Isochebulic acid [19]	C_14_H_12_O_11_	1.78	355.0299	[M–H]^−^	−0.6	**711.0686**, 337.0196, 293.0303, 249.0401, 205.0499
13 ^a,#,♣^	Punicalin α [24]	C_34_H_22_O_22_	1.78	781.0529	[M–H]^−^	0.6	**600.9893**, **448.9792**, **300.9988**
14 ^a,#,♣^	Punicalin β [24]	C_34_H_22_O_22_	1.85	781.0524	[M–H]^−^	−0.4	**600.9888**, **448.9778**, **300.9974**
15 ^a,#,♠^	4-*O*-galloyl-glucose [23]	C_13_H_16_O_10_	1.88	331.06663	[M–H]^−^	−0.6	**663.1405**, 271.0455, 211.0242, **169.0132**, 125.0232
16 ^b,#,♦^	Caffeic acid 3,4-*O*-Di glucuronide [25]	C_21_H_24_O_16_	1.93	531.0993	[M–H]^−^	1.3	**1063.2059**, **355.0307**, 337.0200, 179.0710, 161.0603, 135.0446
17 ^b,#,♠^	4-galloylquinic acid [21]	C_14_H_16_O_10_	2.19	343.0665	[M–H]^−^	0	**709.0888**, **687.1402**, **389.0350**, 191.0535, **169.0132**, 125.0238
18 ^b,#,♣^	Isostrictinin [26]	C_27_H_22_O_18_	2.19	633.0728	[M–H]^−^	0.2	**463.0517**, **300.9981**, 275.0190, **169.0136**, 125.0235
19 ^a,#,♣^	Punicacortein C [20]	C_48_H_28_O_30_	2.33	1083.0585	[M–H]^−^	−0.2	**541.0242**, 1065.0491, 1021.0580, **600.9891**, **499.0722**, **300.9983**, **169.0136**, 125.0235
20 ^a,#,♣^	Punicacortein D [20]	C_48_H_28_O_30_	2.52	1083.0599	[M–H]^−^	1.1	**541.0250**, 1065.0491, 1021.0573, **600.9891**, **499.0722**, **300.9983**, **169.0136**, 125.0235
21 ^a,#,♣^	7′-*O*-methyl chebulate [20]	C_15_H_14_O_11_	2.57	369.0460	[M–H]^−^	0.5	**739.1009**, 351.0338, 325.0563, 307.0460
22 ^a,#,♠^	4-*O*-galloyl-shikimic acid [20]	C_14_H_14_O_9_	2.74	325.0557	[M–H]^−^	−0.9	**651.1190**, 307.0451^−^, 173.0440, **169.0136**, 155.0342, 125.0235
23 ^a,#,♠^	1,4-di-*O*-galloyl-β-D-glucose [23]	C_20_H_20_O_14_	2.78	483.0778	[M–H]^−^	0.6	**967.1592**, **331.0670**, **313.0560**, 211.0242, 193.0137, **169.0135**, 125.0234
24 ^a,#,♣^	Chebumeinin A [27]	C_27_H_26_O_20_	2.78	669.0943	[M–H]^−^	0.6	**691.0753**, **517.0834**, **499.0733**, **337.0200**, 293.0300, 249.0397, 205.0500
25 ^a,#,♠^	5-*O*-galloyl-shikimic acid [20]	C_14_H_14_O_9_	2.92	325.0559	[M–H]^−^	−0.3	**651.1192**, 307.0451, 173.0440, **169.0136**, 155.0342, 125.0235
26 ^a,#,♠^	3-*O*-galloyl-shikimic acid [20]	C_14_H_14_O_9_	3.04	325.0559	[M–H]^−^	−0.3	**651.1185**, 307.0451^−^, 173.0440, **169.0136**, 155.0342, 125.0235
27 ^a,#,♣^	Chebumeinin B [27]	C_27_H_26_O_20_	3.21	669.0939	[M–H]^−^	0.0	**691.0773**, **517.0753**, **499.0724**, **337.0219**, 293.0300, 249.0402, 205.0500
28 ^a,#,♣^	6′-*O*-methyl chebulate [20]	C_15_H_14_O_11_	3.45	369.0453	[M–H]^−^	−1.4	**739.0991**, 351.0316, 325.0558, 307.0438
29 ^a,#,♣^	Valoneic acid dilactone [28]	C_21_H_10_O_13_	3.57	469.0050	[M–H]^−^	−0.6	**939.0164**, 425.0144, 407.0038, **300.9969**, **299.9902**, **169.0137**, 125.0237
30 ^a,#,♠^	2,4-di-*O*-galloyl-β-D-glucose [29]	C_20_H_20_O_14_	3.77	483.0780	[M–H]^−^	1.0	**967.1611**, **331.0666**, **313.0543**, 211.0245, 193.0131, **169.0134**, 125.0236
31 ^a,^*^,♣^	Punicalagin α [20]	C_48_H_28_O_30_	3.84	1083.0583	[M–H]^−^	−0.4	**541.0252**, **781.0526**, **600.9891**, **300.9983**, **169.0136**, 125.0235
32 ^b,#,♠^	3,4-di-*O*-galloyl-β-D-glucose [30]	C_20_H_20_O_14_	3.92	483.0771	[M–H]^−^	−0.8	**967.1619**, **331.0651**, **313.0544**, 271.0446, 211.0243, 193.0128, **169.0134**, 125.0235
33 ^a,#,♣^	Phyllanemblinin D [24]	C_27_H_26_O_20_	4.10	669.0940	[M–H]^−^	0.1	**691.0770**, **517.0822**, **499.0734**, **337.0210**, 293.0293, 249.0396, 205.0501
34 ^b,#,♣^	Rhoipteleanin G [31]	C_48_H_30_O_30_	4.39	1085.0745	[M–H]^−^	0.1	**542.0319**, **783.0687**, **631.0569**, **450.9943**, **300.9985**
35 ^a,#,♣^	Phyllanemblinin F [20]	C_27_H_26_O_20_	4.45	669.0939	[M–H]^−^	0.0	**691.0776**, **499.0734**, **337.0196**, 293.0295, 249.0397, 205.0500
36 ^a,#,♠^	2,6-di-*O*-galloyl-β-D-glucose [32]	C_20_H_20_O_14_	4.59	483.0778	[M–H]^−^	0.6	**967.1616**, **331.0658**, **313.0567**, 271.0459, 211.0241, 193.0138, **169.0134**, 125.0239
37 ^a,#,♠^	4,6-di-*O*-galloyl-β-D-glucose [23]	C_20_H_20_O_14_	4.92	483.0776	[M–H]^−^	0.2	**967.1622**, **331.0672**, **313.0565**, 271.0455, 211.0243, 193.0134, **169.0134**, 125.0236
38 ^a,#,♣^	Strictinin [26]	C_27_H_22_O_18_	5.08	633.0728	[M–H]^−^	0.0	**463.0482**, **300.9982**, 275.0190, **169.0135**, 125.0237
39 ^a,#,♠^	Methyl gallate [20]	C_8_H_8_O_5_	5.41	183.0290	[M–H]^−^	−1.6	**169.0134**, 168.0053, 125.0230, 124.0160
40 ^b,#,♠^	1,2-di-*O*-galloyl-β-D-glucose [33]	C_20_H_20_O_14_	5.69	483.0776	[M–H]^−^	0.2	**967.1614**, **331.0665**, **313.0554**, 271.0451, 211.0243, 193.0136, **169.0134**, 125.0237
41 ^a,#,♠^	2,3-di-*O*-galloyl-β-D-glucose [23]	C_20_H_20_O_14_	6.01	483.0776	[M–H]^−^	0.2	**967.1624**, **331.0657**, **313.0558**, 271.0454, 211.0242, 193.0136, **169.0133**, 125.0237
42 ^a,#,♣^	Phyllanemblinin E [20]	C_27_H_26_O_20_	6.23	669.0933	[M–H]^−^	−0.9	**691.0761**, **499.0725**, **337.0203**, 293.0300, 249.0397, 205.0497
43 ^a,^*^,♣^	Punicalagin β [20]	C_48_H_28_O_30_	6.43	1083.0586	[M–H]^−^	−0.1	**541.0252**, **781.0526**, **600.9891**, **300.9983**, **169.0136**
44 ^a,#,♠^	1,6-di-*O*-galloyl-β-D-glucose [20]	C_20_H_20_O_14_	6.79	483.0780	[M–H]^−^	1.0	**967.1631**, **331.0668**, **313.0558**, 271.0455, 211.0242, 193.0136, **169.0134**, 125.0237
45 ^a,#,♠^	3,6-di-*O*-galloyl-β-D-glucose [20]	C_20_H_20_O_14_	7.21	483.0782	[M–H]^−^	1.4	**967.1636**, **331.0666**, **313.0560**, 271.0458, 211.0243, **169.0135**, 125.0238
46 ^a,#,♣^	Terflavin a [20]	C_48_H_30_O_30_	7.73	1085.0756	[M–H]^−^	1.1	**542.0331**, **783.0696**, **631.0578**, **450.9946**, **300.9987**
47 ^a,#,♦^	Brevifolin carboxylic acid [20]	C_13_H_8_O_8_	7.73	291.0145	[M–H]^−^	1.4	**337.0199**, 247.0232, 203.0342
48 ^a,#,♠^	3,4-di-*O*-galloylshikimic acid [29]	C_21_H_18_O_13_	8.03	477.0672	[M–H]^−^	0.6	**499.0485**, **325.0577**, **307.0452**, **169.0135**, 137.0237, 125.0238
49 ^a,#,♠^	1,3-di-*O*-galloyl-β-D-glucose [32]	C_20_H_20_O_14_	8.21	483.0777	[M–H]^−^	0.4	**967.1615**, **331.0655**, **313.0557**, 271.0454, 211.0240, **169.0136**, 125.0237
50 ^b,#,♣^	Hippomanin A [34]	C_27_H_22_O_18_	8.35	633.0732	[M–H]^−^	0.6	**463.0542**, **300.9984**, 275.0194, **169.0138**, 125.0237
51 ^b,#,♠^	1,2,4-tri-*O*-galloyl-β-D-glucose [35]	C_27_H_24_O_18_	8.57	635.0885	[M–H]^−^	0.2	**317.0391**, **657.0715**, **483.0771**, **465.0676**, **313.0557**, **295.0457**, **169.0135**, 125.0235
52 ^a,#,♣^	Amlaic acid [32]	C_27_H_24_O_19_	9.48	651.0836	[M–H]^−^	0.3	**325.0374**, 633.0739, **481.0624**, **337.0210**, **319.0083**, 275.0190, **169.0135**
53 ^a,#,♣^	Methyl neochebulanin [20](4-*O*-methyl neochebulate-1-*O*-galloyl-glucose)	C_28_H_28_O_20_	9.48	683.1100	[M–H]^−^	0.6	**705.0916**, **341.0500**, **513.0892**, **351.0351**, 307.0457, 263.0559, 219.0293, 204.0395,
54 ^b,#,♦^	Phelligridin J [36]	C_13_H_6_O_8_	9.98	288.9984	[M–H]^−^	1.4	245.0086
55 ^b,#,♠^	1,3,4-tri-*O*-galloyl-β-D-glucose [37]	C_27_H_24_O_18_	10.19	635.0891	[M–H]^−^	1.1	**657.0726**, **317.0398**, **483.0783**, **465.0667**, **313.0562, 295.0447**, **169.0134**, 125.0237
56 ^c,♣^	2-*O*-methyl neochebulate-1-*O*-galloyl-glucose	C_28_H_28_O_20_	10.48	683.1098	[M–H]^−^	0.3	**705.0890**, **341.0493**, **513.0911**, **351.0357**, 307.0466, 263.0541, 219.0296, 204.0395
57 ^a,#,♠^	1,2,6-tri-*O*-galloyl-β-D-glucose [23]	C_27_H_24_O_18_	10.90	635.0886	[M–H]^−^	0.3	**657.0710**, **317.0399**, 483.0784, 465.0669, 313.0558, 295.0454, 169.0134, 125.0239
58 ^b,#,♣^	Carpinusnin [38]	C_41_H_34_O_29_	11.10	989.1116	[M–H]^−^	0.8	**494.0514**, **651.0831**, **481.0624**, **337.0194**
59 ^a,#,♣^	Tercatain [20]	C_34_H_26_O_22_	11.56	785.0833	[M–H]^−^	−0.5	**807.0775**, **392.0367**, **615.0608**, **483.0765**, **463.0505**, **445.0401**, **300.9980**
60 ^a,#,♠^	3,4,6-tri-*O*-galloyl-β-D-glucose [20]	C_27_H_24_O_18_	11.69	635.0884	[M–H]^−^	0	**657.0705**, **317.0396**, **483.0775**, **465.0668**, **313.0557**, **295.0449**, **169.0133**, 125.0237
61 ^b,#,♠^	3,5-di-*O*-galloylshikimic acid [39]	C_21_H_18_O_13_	11.92	477.0671	[M–H]^−^	0.4	**499.0492**, **325.0577**, **307.0466**, **169.0133**, 137.0235, 125.0236
62 ^a,#,♣^	Chebulanin [20]	C_27_H_24_O_19_	12.17	651.0833	[M–H]^−^	−0.2	**325.0369**, 633.0728, **481.0620**, **337.0202**, **319.0089**, 275.0195, **169.0136**, 125.0236
63 ^c,♣^	6-*O*-methyl neochebulate-1-*O*-galloyl-glucose	C_28_H_28_O_20_	12.17	683.1091	[M–H]^−^	−0.7	**705.0886**, **341.0510**, **513.0881**, **351.0343**, 307.0459, 263.0560, 219.0291, 204.0392
64 ^c,♣^	4-galloyl-6-neochebuloyl-2,3-HHDP-glucose	C_41_H_32_O_28_	12.17	971.1006	[M–H]^−^	0.4	**485.0460**, 953.0898, 935.0793, **801.0782**, **669.0914**, **633.0727**, **499.0726**, **463.0513**, 337.0202, **300.9981**
65 ^b,#,♠^	4,5-di-*O*-galloylshikimic acid [39]	C_21_H_18_O_13_	12.89	477.0672	[M–H]^−^	0.6	**499.0485**, **325.0577**, **307.0452**, **169.0135**, 137.0237, 125.0238
66 ^c,♣^	3-*O*-methyl neochebulate-1-*O*-galloyl-glucose	C_28_H_28_O_20_	13.02	683.1088	[M–H]^−^	−1.2	**705.0922**, **341.0499**, **513.0967**, **351.0341**, 307.0438, 263.0558, 219.0274, 204.0385
67 ^c,♣^	1-galloyl-2-neochebuloyl-4,6-HHDP-glucose	C_41_H_32_O_28_	13.29	971.1009	[M–H]^−^	0.7	**485.0450**, 953.0893, 935.0793, **801.0742**, **669.0939**, **633.0729**, **499.0729**, **463.0508**, **337.0195**, **300.9981**
68 ^a,#,♣^	Tellimagrandin I [20]	C_34_H_26_O_22_	13.32	785.0848	[M–H]^−^	1.4	**807.0668**, **392.0373**, **633.0729**, **483.0779**, **463.0504**, **445.0413**, **300.9980**
69 ^a,#,♦^	Urolithin M_5_ [40]	C_13_H_8_O_7_	13.32	275.0195	[M–H]^−^	1.1	**551.0461**, 257.0085, 229.0139, 201.0183
70 ^c,♣^	1-galloyl-3-neochebuloyl-4,6-HHDP-glucose	C_41_H_32_O_28_	13.67	971.1014	[M–H]^−^	1.2	**485.0457**, 953.0887, 935.0786, **801.0779**, **669.0923**, **633.0719**, **499.0723**, **463.0506**, **337.0191**, **300.9980**
71 ^a,^*^,♣^	Corilagin [20]	C_27_H_22_O_18_	14.07	633.0724	[M–H]^−^	−0.6	**463.0514**, **300.9985**, 275.0189, **169.0135**, 125.0235
72 ^a,#,♠^	1,4,6-tri-*O*-galloyl-β-D-glucose [23]	C_27_H_24_O_18_	15.17	635.0880	[M–H]^−^	−0.6	**317.0391**, **657.0696**, **483.0782**, **465.0675**, **313.0558**, **295.0453**, **169.0134**, 125.0236
73 ^b,#,♠^	2,3,4-tri-*O*-galloyl-β-D-glucose [41]	C_27_H_24_O_18_	15.71	635.0881	[M–H]^−^	−0.5	**317.0393**, **657.0696**, **483.0773**, **465.0668**, **313.0555**, **295.0451**, **169.0133**, 125.0238
74 ^c,♣^	1-galloyl-2-neochebuloyl-3,6-HHDP-glucose	C_41_H_32_O_28_	15.96	971.1000	[M–H]^−^	−0.2	**485.0450**, 953.0904, 935.0748, **801.0808**, **669.1053**, **633.0721**, **499.0756**, **463.0502**, **337.0193**, **300.9983**
75 ^b,#,♠^	2,4,6-tri-*O*-galloyl-β-D-glucose [42]	C_27_H_24_O_18_	16.16	635.0881	[M–H]^−^	−0.5	**317.0395**, **657.0709**, **483.0776**, **465.0690**, **313.0558**, **295.0443**, **169.0134**, 125.0240
76 ^b,#,♣^	1,3-di-*O*-galloyl-4,6-HHDP-glucose [43]	C_34_H_26_O_22_	16.25	785.0848	[M–H]^−^	1.4	**807.0668**, **392.0373**, **633.0729**, **483.0779**, **463.0504**, **445.0413**, **300.9980**
77 ^a,#,♠^	1,3,6-tri-*O*-galloyl-β-D-glucose [22]	C_27_H_24_O_18_	16.68	635.0872	[M–H]^−^	−1.9	**317.0391**, **657.0693**, 483.0772, 465.0667, **313.0555**, **295.0449**, **169.0134**, 125.0234
78 ^a,#,♣^	1,3-di-*O*-galloyl-2,4-chebuloyl-D-glucose [44]	C_34_H_28_O_23_	17.10	803.0942	[M–H]^−^	−0.1	**825.0756**, **401.0424**, 785.0836, **633.0723**, 589.0815,533.0569, **483.0198**, **313.0563**
79 ^a,#,♣^	Neochebulagic acid [20](1-galloyl-4-neochebuloyl-3,6-HHDP-glucose)	C_41_H_32_O_28_	17.57	971.0999	[M–H]^−^	−0.3	**485.0457**, 953.0826, 935.0793, **801.0682**, **669.1024**, **633.0735**, **499.0777**, **463.0483**, **337.0194**, **300.9984**
80 ^a,#,♠^	1,2,3,6-tetra-*O*-galloyl-β-D-glucose [20]	C_34_H_28_O_22_	17.62	787.0999	[M–H]^−^	0.6	**809.0811**, **393.0450**, **635.0889**, **617.0783**, **483.0782**, **465.0676**, **447.0562**, **313.0555**, **295.0450**
81 ^a,#,♣^	Tellimagrandin II [18]	C_41_H_30_O_26_	17.82	937.0959	[M–H]^−^	1.3	**468.0425**, **767.0801**, **635.0894**, **465.0661**, **313.0561**, **300.9982**
82 ^b,#,♠^	2,3,6-tri-*O*-galloyl-β-D-glucose [35]	C_27_H_24_O_18_	17.97	635.0879	[M–H]^−^	−0.8	**317.0401**, **657.0699**, **483.0765**, **465.0678**, **313.0568**, **295.0454**, **169.0136**, 125.0237
83 ^c,♣^	1,2,3-tri-*O*-galloyl-4-neochebuloyl-D-glucose	C_41_H_34_O_28_	18.15	973.1159	[M–H]^−^	0.1	**486.0532**, **803.0942**, **635.0881**, **633.0729**, **483.0779**, **465.0667**, **463.0511**, **337.0193**, **300.9985**
84 ^a,#,♣^	Terchebulin [45]	C_48_H_28_O_30_	19.00	1083.0604	[M–H]^−^	1.6	**541.0252**, **781.0580**, **600.9910**, **448.9790**, **300.9985**, **169.0136,** 125.0236
85 ^a,#,♠^	1,2,3-tri-*O*-galloyl-β-D-glucose [23]	C_27_H_24_O_18_	19.24	635.0880	[M–H]^−^	−0.6	**657.0691**, **317.0393**, **483.0773**, **465.0668**, **313.0558**, **295.0443**, **169.0133**, 125.0238
86 ^c,♣^	1-*O*-galloyl-3,4-chebuloyl-2,6-HHDP-D-glucose	C_41_H_30_O_27_	19.65	953.0919	[M–H]^−^	2.4	**476.0416**, **783.0739**, **651.0840**, **633.0735**, **481.0611**, **463.0504**, **337.0199**, **331.0665**, **319.0095**, **300.9988**
87 ^c,♣^	1-galloyl-4-neochebuloyl-2,3-HHDP-glucose	C_41_H_32_O_28_	19.92	971.1003	[M–H]^−^	0.1	**485.0459**, 953.0898, 935.0773, **801.0789**, **669.0975**, **633.0717**, **499.0704**, **463.0513**, **337.0197**, **300.9984**
88 ^a,^*^,♣^	Ellagic acid [20]	C_14_H_6_O_8_	20.04	300.9987	[M–H]^−^	1.0	283.9958, 257.0087, 229.0122, 201.0182, 185.0236, 173.0223, 145.0288, 117.0326
89 ^c,♣^	1-*O*-galloyl-3,4-THDP-2,6-HHDP-D-glucose	C_40_H_30_O_26_	20.13	925.0954	[M–H]^−^	0.8	**462.0426**, **773.0887**, **755.0728**, **633.0735, 463.0518**, **300.9986**
90 ^c,♣^	1-*O*-galloyl-3,6-chebuloyl-2,4-HHDP-D-glucose	C_41_H_30_O_27_	20.27	953.0895	[M–H]^−^	−0.1	**476.0406**, **783.0743**, **651.0848**, **633.0744**, **481.0629**, **463.0519**, **337.0198**, **331.0643**, **319.0092**, **300.9991**
91 ^b,#,♣^	Punicafolin [38]	C_41_H_30_O_26_	20.29	937.0961	[M–H]^−^	1.5	**468.0435**, **767.0743**, **635.0884**, **465.0688**, **313.0545**, **300.9987**
92 ^c,♣^	1,6-di-*O*-galloyl-2,4-chebuloyl-D-glucose	C_34_H_28_O_23_	20.37	803.0957	[M–H]^−^	1.7	**401.0431**, **825.0756**, 785,0836, **633.0723**, 589.0815, 533.0569, **483.0198**, **313.0563**
93 ^b,#,♣^	Phyllantusiin C [30]	C_40_H_30_O_26_	20.37	925.0955	[M–H]^−^	−0.3	**462.0449**, **773.0849**, **755.0762**, **633.0731**, **463.0514**, **300.9985**
94 ^a,♣^	1,3,6-tri-*O*-galloyl-4-neochebuloyl-D-glucose [29]	C_41_H_34_O_28_	20.50	973.1162	[M–H]^−^	0.4	**486.0536**, **803.0956**, **635.0879**, **633.0724**, **483.0755**, **465.0685**, **463.0514**, **337.0192**, **300.9984**
95 ^c,♣^	1-*O*-galloyl-4,6-chebuloyl-2,3-HHDP-D-glucose	C_41_H_30_O_27_	20.60	953.0893	[M–H]^−^	−0.3	**476.0412**, **783.0712**, **651.0827**, **633.0726**, **481.0653**, **463.0526**, **337.0191**, **331.0658**, **319.0093**, **300.9987**
96 ^c,♣^	1-*O*-galloyl-3,6-THDP-2,4-HHDP-D-glucose	C_40_H_30_O_26_	20.77	925.0959	[M–H]^−^	1.3	**462.0430**, **773.0862**, **755.0744**, **633.0735**, **463.0521**, **300.9988**
97 ^b,#,♣^	Davidiin [46]	C_41_H_30_O_26_	20.86	937.0964	[M–H]^−^	1.8	**468.0435**, **767.0731**, **635.0885**, **465.0673**, **313.0563**, **300.9987**
98 ^c,♣^	3,6-di-*O*-galloyl-2,4-chebuloyl-D-glucose	C_34_H_28_O_23_	20.90	803.0961	[M–H]^−^	2.2	**825.0776**, **401.0432**, 785.0864, **633.0743**, 589.0851, 533.0582, **483.0163**, **313.0558**
99 ^c,♠^	3,4,5-tri-*O*-galloyl shikimic acid	C_28_H_22_O_18_	20.90	629.0789	[M–H]^−^	1.6	**477.0655**, **459.0561**, **325.0501**, **307.0443**, **289.0357**, **169.0138**
100 ^c,♣^	1,2,6-tri-*O*-galloyl-4-neochebuloyl-D-glucose	C_41_H_34_O_28_	20.94	973.1168	[M–H]^−^	1.0	**486.0546**, **803.0958**, **635.0894**, **633.0729**, **483.0772**, **465.0677**, **463.0529**, **337.0198**, **300.9986**
101 ^c,♣^	2-galloyl-3-neochebuloyl-4,6-HHDP-glucose	C_41_H_32_O_28_	20.94	971.1013	[M–H]^−^	1.1	**485.0463**, 953.0900, 935.0801, **801.0813**, **669.0943**, **633.0731**, **499.0749**, **463.0515**, **337.0198**, **300.9986**
102 ^a,#,♣^	Eschweilenol C [20]	C_20_H_16_O_12_	21.01	447.0573	[M–H]^−^	2.0	**895.1224**, **300.9984**
103 ^c,♣^	1-*O*-galloyl-4,6-THDP-2,3-HHDP-D-glucose	C_40_H_30_O_26_	21.07	925.0956	[M–H]^−^	1.0	**462.0424**, **773.0867**, **755.0684**, **633.0731**, **463.0518**, **300.9983**
104 ^c,♣^	1,3,4-tri-*O*-galloyl-2,6-HHDP-glucose	C_41_H_30_O_26_	21.10	937.0964	[M–H]^−^	1.8	**468.0437**, **767.0753**, **635.0886**, **465.0679**, **313.0557**, **300.9991**
105 ^a,^*^,♣^	Chebulagic acid [22]	C_41_H_30_O_27_	21.15	953.0896	[M–H]^−^	1.7	**476.0415**, **783.0693**, **651.0840**, **633.0735**, **481.0627**, **463.0518**, **337.0198**, **331.0667**, **319.0092**, **300.9990**
106 ^c,♣^	2,3,6-tri-*O*-galloyl-4-neochebuloyl-D-glucose	C_41_H_34_O_28_	21.29	973.1172	[M–H]^−^	1.4	**486.0541**, **803.0955**, **635.0891**, **633.07235**, **483.0763**, **465.0677**, **463.0523**, **337.0198**, **300.9992**
107 ^b,#,♣^	Pterocarinin C [47]	C_41_H_30_O_26_	21.34	937.0959	[M–H]^−^	1.3	**468.0432**, **767.0742**, **635.0887**, **465.0670**, **313.0559**, **300.9989**
108 ^a,#,♠^	1,2,3,4-tetra-*O*-galloyl-β-D-glucose [23]	C_34_H_28_O_22_	21.35	787.0999	[M–H]^−^	0.6	**809.0834**, **393.0457**, **635.0889**, **617.0782**, **483.0770**, **465.0676**, **447.0555**, **313.0556**, **295.0450**
109 ^c,♣^	1-galloyl-6-neochebuloyl-2,3-HHDP-glucose	C_41_H_32_O_28_	21.39	971.1014	[M–H]^−^	1.2	**485.0464**, 953.0909, 935.0782, **801.0809**, **669.1044**, **633.0738**, **499.0724**, **463.0503, 337.0197**, **300.9990**
110 ^b,#,♣^	1,6-di-*O*-galloyl-2,3-HHDP-glucose [48]	C_34_H_26_O_22_	21.47	785.0848	[M–H]^−^	1.4	**807.0668**, **392.0373**, **633.0729**, **483.0779**, **463.0504**, **445.0413**, **300.9980**
111 ^c,♣^	1,2,3-tri-*O*-galloyl-4-methyl neochebuloyl-glucose	C_42_H_36_O_28_	21.74	987.1318	[M–H]^−^	0.3	**493.0620**, **817.1097**, **635.0881**, **465.0668**, **351.0352**, **295.0450**
112 ^a,#,♠^	1,2,4,6-tetra-*O*-galloyl-β-D-glucose [23]	C_34_H_28_O_22_	21.85	787.0994	[M–H]^−^	0.0	**809.0809**, **393.0447**, **635.0881**, **617.0773**, **483.0768**, **465.0674**, **447.0560**, **313.0555**, **295.0451**
113 ^a,#,♣^	1-*O*-galloyl-3,6-HHDP-4-6′ methyl neochebuloyl-glucose [49]	C_42_H_34_O_28_	21.94	985.1156	[M–H]^−^	−0.2	**492.0533**, **815.0938**, **683.0846**, **633.0719**, **513.0870**, **463.0513**, **351.0342**, **300.9983**
114 ^a,#,♠^	1,3,4,6-tetra-*O*-galloyl-β-D-glucose [20]	C_34_H_28_O_22_	22.00	787.0995	[M–H]^−^	0.1	**809.0819**, **393.0447**, **635.0874**, **617.0775**, **483.0771**, **465.0668**, **447.0558**, **313.0556**, **295.0449**
115 ^c,♣^	2-*O*-galloyl-3,6-HHDP-4-6′ methyl neochebuloyl-glucose	C_42_H_34_O_28_	22.13	985.1154	[M–H]^−^	−0.4	**492.0534**, **815.0939**, **683.0846**, **633.0718**, **513.0867**, **463.0512**, **351.0346**, **300.9983**
116 ^c,♣^	1-*O*-galloyl-2,3-THDP-4,6-HHDP-D-glucose	C_40_H_30_O_26_	22.20	925.0941	[M–H]^−^	−0.6	**462.0423**, **773.0825**, **755.0762**, **633.0716**, **463.0508**, **300.9982**
117 ^a,#,♣^	1,2-di-*O*-galloyl-4,6-HHDP-glucose [50]	C_34_H_26_O_22_	22.29	785.0833	[M–H]^−^	−0.5	**807.0775**, **392.0367**, **615.0608**, **483.0765**, **463.0505**, **445.0401**, **300.9980**
118 ^c,♣^	2-galloyl-4-neochebuloyl-3,6-HHDP-glucose	C_41_H_32_O_28_	22.49	971.1006	[M–H]^−^	0.4	**485.0472**, 953.0893, 935.0806, **801.0945**, **669.0826**, **633.0731**, **499.0716**, **463.0505**, **337.0193**, **300.9982**
119 ^c,♣^	1-*O*-galloyl-2,6-chebuloyl-3,4-HHDP-D-glucose	C_41_H_30_O_27_	22.65	953.0883	[M–H]^−^	−1.4	**476.0383**, **783.0655**, **651.0851**, **633.0722**, **481.0618**, **463.0501**, **337.0194**, **331.0634**, **319.0079**, **300.9979**
120 ^b,#,♠^	2,3,4,6-tetra-*O*-galloyl-β-D-glucose [51]	C_34_H_28_O_22_	22.66	787.0986	[M–H]^−^	−1.0	**809.0819**, **393.0447**, **635.0871**, **617.0767**, **483.0764**, **465.0662**, **447.0559**, **313.0554**, **295.0446**
121 ^c,♣^	6-galloyl-4-neochebuloyl-2,3-HHDP-glucose	C_41_H_32_O_28_	22.84	971.0999	[M–H]^−^	−0.3	**485.0458**, 953.0894, 935.0800, **801.0879**, **669.0791**, **633.0726**, **499.0721**, **463.0498**, **337.0190**, **300.9981**
122 ^c,♣^	1,2,3-tri-*O*-galloyl-4,6-neochebuloyl-glucose	C_41_H_32_O_27_	23.03	955.1048	[M–H]^−^	−0.5	**477.0480**, **785.0828**, **617.0756**, **465.0661**, **447.0555**, **337.0194**
123 ^c,♣^	1-*O*-galloyl-2,3-chebuloyl-4,6-HHDP-D-glucose	C_41_H_30_O_27_	23.76	953.0882	[M–H]^−^	−1.5	**476.0395**, **783.0699**, **651.0839**, **633.0712**, **481.0641**, **463.0492**, **337.0190**, **331.0685**, **319.0087**, **300.9981**
124 ^c,♣^	1,2,4-tri-*O*-galloyl-3,6-neochebuloyl-glucose	C_41_H_32_O_27_	23.82	955.1045	[M-H]^−^	−0.8	**477.0481**, **785.0828**, **617.0765**, **465.0669**, **447.0562**, **337.0189**
125 ^a,#,♣^	1,3,6-tri-*O*-galloyl-4-methyl neochebuloyl-glucose [20]	C_42_H_36_O_28_	23.93	987.1302	[M–H]^−^	1.1	**493.0611**, **817.1092**, **635.0872**, **465.0662**, **351.0343**, **295.0445**
126 ^c,♣^	1,2,6-tri-*O*-galloyl-4-methyl neochebuloyl-glucose	C_42_H_36_O_28_	24.36	987.1313	[M–H]^−^	−0.2	**493.5631**, **817.1094**, **635.0876**, **465.0673**, **351.0338**, **295.0440**
127 ^a,^*^,♣^	Chebulinic acid [20](1,3,6-tri-*O*-galloyl-2,4-chebuloyl-glucose)	C_41_H_32_O_27_	24.57	955.1054	[M–H]^−^	0.1	**477.0486**, **785.0839**, **635.0873**, 617.0771, **465.0668**, **447.0558**, 337.0190
128 ^c,♣^	2,3,6-tri-*O*-galloyl-4-methyl neochebuloyl-glucose	C_42_H_36_O_28_	25.47	987.1324	[M–H]^−^	0.9	**493.0623**, **817.1107**, **635.0883**, **465.0674**, 351.0348, **295.0458**
129 ^c,♣^	1,4,6-tri-*O*-galloyl-2,3-neochebuloyl-glucose	C_41_H_32_O_27_	25.47	955.1041	[M–H]^−^	−1.3	**477.0491**, **785.0850**, **617.0781**, **465.0674**, **447.0564**, **337.0197**
130 ^a,^*^,♠^	1,2,3,4,6-penta-*O*-galloyl-β-D-glucose [22]	C_41_H_32_O_26_	26.73	939.1113	[M–H]^−^	1.0	**469.0515**, **787.1005**, **769.0900**, **617.0784**, **599.0676**, **465.0677**, **447.0568**, **429.0460**, **313.0565**, **295.0456**, **277.0352**, **259.0245**, **169.0136**, 125.0237
131 ^a,#,♣^	Terchebin [52]	C_41_H_30_O_27_	28.19	953.0901	[M–H]^−^	0.5	935.0799, 917.0695, **617.0781**, **465.0671**, **316.9932**, **295.0452**
132 ^a,#,♣^	4-*O*-(4″-*O*-galloyl-α-rhamnopyranosyl) ellagic acid [49]	C_27_H_20_O_16_	28.94	599.0673	[M–H]^−^	0.0	**1199.1431**, **621.0486**, **447.0560**, **429.0441**, **300.9985**
133 ^c,♣^	1-*O*-galloyl-2,6-THDP-3,4-HHDP-D-glucose	C_40_H_30_O_26_	29.04	925.0959	[M–H]^−^	1.3	**462.0432**, **773.0815**, **755.0752**, **633.0731**, **463.0506**, **300.9982**
134 ^a,#,♣^	4-*O*-(2″,3″-di-*O*-galloyl-α-L-rhamnosyl) ellagic acid [20]	C_34_H_24_O_20_	29.31	751.0782	[M–H]^−^	−0.1	**375.0342**, **599.0668**, **581.0553**, **449.0718**, **411.0366**, **300.9985**
135 ^b,#,♣^	Nupharin A [53]	C_41_H_30_O_26_	29.35	937.0955	[M–H]^−^	0.9	**468.0433**, **767.0732**, **635.0894**, **465.0674**, **313.0573**, **300.9988**
136 ^c,♠^	1-*O*-cinnamoyl-6-*O*-galloyl-glucose	C_22_H_22_O_11_	29.39	461.1084	[M–H]^−^	0	313.0558, **169.0135**, 147.0436
137 ^a,#,♣^	4-*O*-(2″,4″-di-*O*-galloyl-α-L-rhamnosyl) ellagic acid [20]	C_34_H_24_O_20_	29.42	751.0782	[M–H]^−^	−0.1	**375.0342**, **599.0668**, **581.0553**, **449.0718**, **411.0366**, **300.9985**
138 ^a,#,♣^	4-*O*-(3″,4″-di-*O*-galloyl-α-rhamnopyranosyl) ellagic acid [49]	C_34_H_24_O_20_	29.50	751.0776	[M–H]^−^	−0.9	**375.0338**, **599.0660**, **581.0549**, **449.0713**, **411.0292**, **300.9977**
139 ^c,♥^	Arjungenin-24-*O*-glucoheptonic acid	C_37_H_60_O_13_	29.59	711.3952	[M–H]^−^	−0.6	**503.3379**
140 ^b,#,♠^	1-*O*-galloyl-2-*O*-cinnamoyl-glucose [54]	C_22_H_22_O_11_	29.73	461.1092	[M–H]^−^	1.7	**923.2247**, 313.0561, **169.0133**, 147.0443
141 ^a,#,♥^	Quercotriterpenoside I [55]	C_43_H_62_O_15_	29.73	817.4018	[M–H]^−^	1.0	**655.3484**, **503.3366**
142 ^b,#,♠^	1-*O*-galloyl-6-*O*-cinnamoyl-glucose [54]	C_22_H_22_O_11_	29.81	461.1077	[M–H]^−^	−1.5	313.0562, **169.0132**, 147.0443
143 ^a,#,♠^	1,2-*O*-galloyl-6-*O*-cinnamoyl-glucose [20]	C_29_H_26_O_15_	29.82	613.1190	[M–H]^−^	−0.5	465.0682, **461.1077**, 313.0560, **169.0131**, 147.0443
144 ^b,#,♠^	1-*O*-cinnamoyl-2-*O*-galloyl-glucose [56]	C_22_H_22_O_11_	29.95	461.10978	[M–H]^−^	−1.3	313.0549, **169.0132**, 147.0434
145 ^c,♥^	Madecassic acid-24-galloyl-28-glucose	C_43_H_62_O_15_	30.13	817.4008	[M–H]^−^	−0.2	**655.3470**, **503.3369**
146 ^b,#,♠^	1-*O*-cinnamoyl-2,6-*O*-galloyl-glucose [57]	C_29_H_26_O_15_	30.19	613.1190	[M–H]^−^	−0.5	465.0667, **461.1085**, 313.0558, **169.0131**, 147.0441
147 ^a,#,♥^	Terminolic acid-24-galloyl-28-glucose [56]	C_43_H_62_O_15_	30.27	817.3998	[M–H]^−^	−1.5	**655.3473**, **503.3361**
148 ^c,♥^	Rotundic acid-24-galloyl-28-glucose	C_43_H_62_O_14_	30.30	801.4066	[M–H]^−^	0.6	**639.3536**, **487.3401**
149 ^c,♥^	Madecassic acid-24-*O*-glucoheptonic acid	C_37_H_60_O_13_	30.34	711.3954	[M–H]^−^	−0.3	**503.3372**
150 ^c,♥^	Rotundic acid-24-*O*-glucoheptonic acid	C_37_H_60_O_12_	30.37	695.3983	[M–H]^−^	−3.5	**487.3413**
151 ^c,♥^	Asiatic acid-24-galloyl-28-glucose	C_43_H_62_O_14_	30.39	801.4073	[M–H]^−^	1.5	**639.3538**, **487.3424**
152 ^a,#,♠^	1,6-*O*-galloyl-2-*O*-cinnamoyl-glucose [20]	C_29_H_26_O_15_	30.39	613.1200	[M–H]^−^	1.1	465.0645, **461.1104**, 313.0572, **169.0134**, 147.0451
153 ^c,♥^	Terminolic acid-24-*O*-glucoheptonic acid	C_37_H_60_O_13_	30.43	711.3954	[M–H]^−^	−0.3	**503.3377**
154 ^c,♥^	Asiatic acid-24-*O*-glucoheptonic acid	C3_7_H_60_O_12_	30.47	695.4011	[M–H]^−^	0.6	**487.3428**
155 ^c,♥^	Arjunolic acid-24-galloyl-28-glucose	C_43_H_62_O_14_	30.51	801.4060	[M–H]^−^	1–0.1	**639.3539**, **487.3427**
156 ^c,♥^	Arjunolic acid-24-*O*-glucoheptonic acid	C_37_H_60_O_12_	30.57	695.4019	[M–H]^−^	0.6	**487.3432**
157 ^a,#,♠^	1,2,3-tri-*O*-galloyl-6-*O*-cinnamoyl-β- D-glucose [20]	C_36_H_30_O_19_	30.57	765.1310	[M–H]^−^	0.9	**811.4279**, **787.1131**, 635.0932, 617.0841, **613.1192, 595.1093**, **443.0975**
158 ^a,#,♥^	Arjungenin [40]	C_30_H_48_O_6_	31.12	503.3384	[M–H]^−^	2.2	**1007.6838**, **549.3438**
159 ^a,#,♥^	Madecassic acid [28]	C_30_H_48_O_6_	31.56	503.3387	[M–H]^−^	2.8	**1007.6841**, **549.3439**
160 ^a,#,♥^	23-galloyl-arjunolic acid [58]	C_37_H_52_O_9_	31.71	639.3547	[M–H]^−^	2.2	**487.3378**
161 ^a,#,♥^	Terminolic acid [55]	C_30_H_48_O_6_	31.77	503.3387	[M–H]^−^	2.8	**1007.6847**, **549.3439**
162 ^b,#,♥^	Rotundic acid [59]	C_30_H_48_O_5_	31.90	487.3437	[M–H]^−^	2.9	**975.6937**
163 ^a,#,♥^	Asiatic acid [32]	C_30_H_48_O_5_	32.09	487.3438	[M–H]^−^	3.1	**975.6949**
164 ^a,#,♥^	Arjunolic acid [18]	C_30_H_48_O_5_	32.35	487.3434	[M–H]^−^	2.3	**975.6979**

## Data Availability

The datasets used and/or analyzed during the current study are available from the corresponding author on reasonable request.

## Supplementary Materials

The following supporting information can be downloaded at: https://www.mdpi.com/article/10.3390/molecules30112451/s1, Table S1: The LogP and CLogP values of the chemical components in *T. chebula* were identified by UPLC-Q-TOF/MS.
