# Supplementary material for: An Innovative Strategy for Untargeted Mass Spectrometry Data Analysis: Rapid Chemical Profiling of the Medicinal Plant Terminalia chebula Using Ultra-High-Performance Liquid Chromatography Coupled with Q/TOF Mass Spectrometry–Key Ion Diagnostics–Neutral Loss Filtering
Source: Molecules. 2025 Jun 3;30(11):2451. doi: 10.3390/molecules30112451 (PMC12157847; doi:10.3390/molecules30112451)
Supplement: Supplementary file 1 [file molecules-30-02451-s001.zip › molecules-3602481-supplementary.pdf]

An Innovative Strategy for Untargeted Mass Spectrometry Data Analysis: Rapid Chemical Profiling of the Medicinal Plant *Terminalia chebula* Using Ultra-high Performance Liquid Chromatography coupled with Q/TOF mass spectrometry-Key Ion Diagnostics-Neutral Loss Filtering

Yu Jia [1], Xinyan Zhao [2], Yuqi He [2], Yi Zhang[1*], Ce Tang[1*]

[1] School of Ethnic Medicine, Chengdu University of Traditional Chinese Medicine, Chengdu 611137, China; 13515094@qq.com (Y.J.)

2 School of Pharmacy, Chengdu University of Traditional Chinese Medicine, Chengdu 611137, China; 2628663829@qq.com (X.Z.); 1838258569@qq.com (Y.H.)

[*]Corresponding: zhangyi@cdutcm.edu.cn (Y.Z.); tangce@cdutcm.edu.cn (C.T.)

Table S1 The LogP and CLogP values of the chemical components in *T. chebula* were identified by UPLC-Q-TOF/MS

No.	Identification	LogP	CLogP
1[b]	3-O-Galloyl-glucose [17]	-1.42	-1.080
2[a]	Shikimic acid [21]	-1.57	-1.162
3[a]	Neochebulic acid [22]	-0.92	-1.297
4[a]	Chebulic acid [19]	-0.92	-1.297
5[b]	3-Galloylquinic acid [23]	-1.48	-2.253
6[a]	1-O-Galloyl-glucose [18]	-1.42	-1.174
7[a]	6-O-Galloyl-glucose [19]	-1.42	-1.422
8[a]	Gallic acid [18]	0.42	0.425
9[a]	2-O-Galloyl-glucose [20]	-1.42	-1.719
10[a]	Gemin D [19]	0.14	-0.003
11[b]	5-Galloylquinic acid [23]	-1.48	-2.253
12[a]	Isochebulic acid [22]	-1.18	-1.700
13[a]	Punicalin α [24]	-0.13	-1.192
14[a]	Punicalin β [24]	-0.13	-1.192
15[a]	4-O-Galloyl-glucose [20]	-1.42	-2.359
16[b]	Caffeic acid 3,4-O-Di Glucuronide [25]	-2.61	-3.226
17[b]	4-Galloylquinic acid [23]	-1.48	-1.877
18[b]	Isostrictinin [26]	-1.48	0.475
19[a]	Punicacortein C [19]	/	0.480
20[a]	Punicacortein D [19]	/	0.480
21[a]	7'-O-methyl chebulate [19]	-0.66	-0.822
22[a]	4-*O*-Galloyl-shikimic acid [19]	-0.61	-0.029
23[a]	1,4-Di-O-galloyl-β-D-glucose [20]	-0.46	-1.319
24[a]	Chebumeinin A [28]	-1.8	-1.990
25[a]	5-*O*-Galloyl-shikimic acid [19]	-0.61	-0.620
26[a]	3-*O*-Galloyl-shikimic acid [19]	-0.61	-0.620
27[a]	Chebumeinin B [28]	-1.8	-2.696
28[a]	6'-O-methyl chebulate [19]	-0.66	-0.822
29[a]	Valoneic acid dilactone [29]	1.37	0.350
30[a]	2,4-Di-O-galloyl-β-D-glucose [33]	-0.46	-1.209
31[a]	Punicalagin α [19]	/	1.239
32[b]	3,4-Di-O-galloyl-β-D-glucose [32]	-0.46	-1.117
33[a]	Phyllanemblinin D [24]	-1.8	-1.331
34[b]	Rhoipteleanin G [34]	/	0.559
35[a]	Phyllanemblinin F [19]	-1.8	-1.931
36[a]	2,6-Di-O-galloyl-β-D-glucose [30]	-0.46	-0.928
37[a]	4,6-Di-O-galloyl-β-D-glucose [20]	-0.46	-0.912
38[a]	Strictinin [26]	0.14	-0.272
39[a]	Methyl gallate [19]	0.69	0.931
40[b]	1,2-Di-O-galloyl-β-D-glucose [31]	-0.46	-0.679

41[a]	2,3-Di-O-galloyl-β-D-glucose [20]	-0.46	-0.477
42[a]	Phyllanemblinin E [19]	-1.8	-2.637
43[a]	Punicalagin β [19]	/	1.239
44[a]	1,6-Di-O-galloyl-β-D-glucose [19]	-0.46	-0.382
45[a]	3,6-Di-O-galloyl-β-D-glucose [19]	-0.46	-0.289
46[a]	Terflavin a [19]	/	0.975
47[a]	Brevifolin carboxylic acid [19]	-1.38	-0.742
48[a]	3,4-Di-O-galloylshikimic acid [33]	0.35	0.623
49[a]	1,3-Di-O-galloyl-β-D-glucose [30]	-0.46	-0.040
50[b]	Hippomanin A [27]	0.14	-0.162
51[b]	1, 2, 4-Tri-O-galloyl-β-D-glucose [38]	0.5	-0.169
52[a]	Amlaic acid [30]	-1.27	-2.893
53[a]	Methyl neochebulanin [19]<br>(4-O-methyl neochebulate-1-O-Galloyl-glucose)	-1.54	-2.162
54[b]	Phelligridin J [40]	-0.96	0.830
55[b]	1, 3, 4-Tri-O-galloyl-β-D-glucose [37]	0.5	-0.077
56[c]	2-O-methyl neochebulate-1-O-Galloyl-glucose	-1.54	-1.509
57[a]	1, 2, 6-Tri-O-galloyl-β-D-glucose [20]	0.5	0.112
58[b]	Carpinusnin [41]	/	-3.071
59[a]	Tercatain [19]	1.1	1.102
60[a]	3, 4, 6-Tri-O-galloyl-β-D-glucose [19]	0.5	0.330
61[b]	3,5-Di-O-galloylshikimic acid [35]	0.35	0.579
62[a]	Chebulanin [19]	-1.52	-2.954
63[c]	6-O-methyl neochebulate-1-O-Galloyl-glucose	-1.54	-1.457
64[c]	4-galloyl-6-neochebuloyl-2,3-HHDP-glucose	/	-0.096
65[b]	4,5-Di-O-galloylshikimic acid [35]	0.35	0.623
66[c]	3-O-methyl neochebulate-1-O-Galloyl-glucose	-1.54	-0.856
67[c]	1-galloyl-2-neochebuloyl-4,6-HHDP-glucose	/	-0.426
68[a]	Tellimagrandin I [19]	1.1	1.255
69[a]	Urolithin M_5 [45]	1.21	0.734
70[c]	1-galloyl-3-neochebuloyl-4,6-HHDP-glucose	/	-0.424
71[a]	Corilagin [19]	0.14	0.417
72[a]	1, 4, 6-Tri-O-galloyl-β-D-glucose [20]	0.5	0.128
73[b]	2, 3, 4-Tri-O-galloyl-β-D-glucose [39]	0.5	0.142
74[c]	1-galloyl-2-neochebuloyl-3,6-HHDP-glucose	/	-0.388
75[b]	2, 4, 6-Tri-O-galloyl-β-D-glucose [36]	0.5	0.238
76[b]	1, 3-Di-O-galloyl-4,6-HHDP-glucose [42]	1.1	1.036
77[a]	1, 3, 6-Tri-O-galloyl-β-D-glucose [18]	0.5	0.751
78[a]	1,3-Di-O-galloyl-2,4-chebuloyl-D-glucose [46]	-0.56	-1.613
79[a]	Neochebulagic acid [19]<br>(1-galloyl-4-neochebuloyl-3,6-HHDP-glucose)	/	-0.385
80[a]	1,2,3,6-Tetra-O-galloyl-β-D-glucose [19]	1.46	1.354
81[a]	Tellimagrandin II [21]	2.06	2.295

82[b]	2, 3, 6-Tri-O-galloyl-β-D-glucose [38]	0.5	0.314
83[c]	1,2,3-Tri-O-galloyl-4-neochebuloyl-D-glucose	/	-0.244
84[a]	Terchebulin [47]	/	2.091
85[a]	1, 2, 3-Tri-O-galloyl-β-D-glucose [20]	0.5	0.563
86[c]	1-O-galloyl-3,4-chebuloyl-2,6-HHDP-D-glucose	0.29	-0.933
87[c]	1-galloyl-4-neochebuloyl-2,3-HHDP-glucose	/	-0.327
88[a]	Ellagic acid [19]	1.05	0.376
89[c]	1-O-galloyl-3,4-THDP-2,6-HHDP-D-glucose	0.22	-0.882
90[c]	1-O-galloyl-3,6-chebuloyl-2,4-HHDP-D-glucose	0.29	-1.207
91[b]	Punicafolin [41]	2.06	2.427
92[c]	1,6-Di-O-galloyl-2,4-chebuloyl-D-glucose	-0.56	-1.715
93[b]	Phyllantusiin C [32]	0.22	-0.882
94[a]	1,3,6-Tri-O-galloyl-4-neochebuloyl-D-glucose [33]	/	-0.056
95[c]	1-O-galloyl-4,6-chebuloyl-2,3-HHDP-D-glucose	0.29	-1.029
96[c]	1-O-galloyl-3,6-THDP-2,4-HHDP-D-glucose	0.22	-0.882
97[b]	Davidiin [49]	2.06	2.415
98[c]	3,6-Di-O-galloyl-2,4-chebuloyl-D-glucose	-0.56	-1.320
99[c]	3,4,5-Tri-O-galloyl shikimic acid	1.31	1.930
100[c]	1,2,6-Tri-O-galloyl-4-neochebuloyl-D-glucose	/	-0.040
101[c]	2-galloyl-3-neochebuloyl-4,6-HHDP-glucose	/	-0.313
102[a]	Eschweilenol C [19]	-0.33	-2.256
103[c]	1-O-galloyl-4,6-THDP-2,3-HHDP-D-glucose	0.22	-0.882
104[c]	1,3,4-Tri-O-galloyl-2,6-HHDP-glucose	2.06	2.415
105[a]	Chebulagic acid [18]	0.29	-0.958
106[c]	2,3,6-Tri-O-galloyl-4-neochebuloyl-D-glucose	/	0.163
107[b]	Pterocarinin C [50]	2.06	2.607
108[a]	1,2,3,4-Tetra-O-galloyl-β-D-glucose [20]	1.46	1.182
109[c]	1-galloyl-6-neochebuloyl-2,3-HHDP-glucose	/	-0.283
110[b]	1, 6-Di-O-galloyl-2,3-HHDP-glucose [43]	1.1	1.266
111[c]	1,2,3-Tri-O-galloyl-4-methyl neochebuloyl-glucose	/	0.231
112[a]	1,2,4,6-Tetra-O-galloyl-β-D-glucose [20]	1.46	1.278
113[a]	1-O-galloyl-3,6-HHDP-4-6′ methyl neochebuloyl-glucose [52]	/	0.031
114[a]	1,3,4,6-Tetra-O-galloyl-β-D-glucose [19]	1.46	1.370
115[c]	2-O-galloyl-3,6-HHDP-4-6′ methyl neochebuloyl-glucose	/	0.316
116[c]	1-O-galloyl-2,3-THDP-4,6-HHDP-D-glucose	0.22	-1.034
117[a]	1, 2-Di-O-galloyl-4,6-HHDP-glucose [44]	1.1	0.878
118[c]	2-galloyl-4-neochebuloyl-3,6-HHDP-glucose	/	-0.100
119[c]	1-O-galloyl-2,6-chebuloyl-3,4-HHDP-D-glucose	0.29	-1.000
120[b]	2,3,4,6-Tetra-O-galloyl-β-D-glucose [53]	1.46	1.589
121[c]	6-galloyl-4-neochebuloyl-2,3-HHDP-glucose	/	-0.034
122[c]	1,2,3-Tri-O-galloyl-4,6-neochebuloyl-glucose	/	-0.624
123[c]	1-O-galloyl-2,3-chebuloyl-4,6-HHDP-D-glucose	0.29	-0.904
124[c]	1,2,4-Tri-O-galloyl-3,6-neochebuloyl-glucose		-0.493

125[a]	1,3,6-Tri-O-galloyl-4-methyl neochebuloyl-glucose [19]	/	0.419
126[c]	1,2,6-Tri-O-galloyl-4-methyl neochebuloyl-glucose	/	0.435
127[a]	Chebulinic acid [19] (1,3,6-Tri-O-galloyl-2,4-chebuloyl-glucose)	/	-0.166
128[c]	2,3,6-Tri-O-galloyl-4-methyl neochebuloyl-glucose	/	0.638
129[c]	1,4,6-Tri-O-galloyl-2,3-neochebuloyl-glucose	/	-0.213
130[a]	1,2,3,4,6-Penta-O-galloyl-β-D-glucose [18]	2.42	2.629
131[a]	Terchebin [48]	0.87	-2.224
132[a]	4-O-(4″-O-Galloyl-α-rhamnopyranosyl) ellagic acid [52]	1.02	-0.431
133[c]	1-O-galloyl-2,6-THDP-3,4-HHDP-D-glucose	0.22	-1.948
134[a]	4-O-(2″,3″-Di-O-galloyl-α-L-rhamnosyl) ellagic acid [19]	1.98	0.796
135[b]	Nupharin A [51]	2.06	2.487
136[c]	1-O-cinnamoyl-6-O-galloyl-glucose	1.05	1.331
137[a]	4-O-(2″,4″-Di-O-galloyl-α-L-rhamnosyl) ellagic acid [19]	1.98	0.719
138[a]	4-O-(3″,4″-Di-O-galloyl-α-rhamnopyranosyl) ellagic acid [52]	1.98	0.812
139[c]	Arjungenin-24-O-glucoheptonic acid	/	2.553
140[b]	1-O-galloyl-2-O-cinnamoyl-glucose [54]	1.05	1.056
141[a]	Quercotriterpenoside I [56]	/	4.744
142[b]	1-O-galloyl-6-O-cinnamoyl-glucose [54]	1.05	1.243
143[a]	1,2-O-galloyl-6-O-cinnamoyl-glucose [19]	2.01	1.737
144[b]	1-O-cinnamoyl-2-O-galloyl-glucose [55]	1.05	1.034
145[c]	Madecassic acid-24-galloyl-28-glucose	/	4.126
146[b]	1-O-cinnamoyl-2,6-O-galloyl-glucose [57]	2.01	1.825
147[a]	Terminolic acid-24-galloyl-28-glucose [56]	/	4.126
148[c]	Rotundic acid-24-galloyl-28-glucose	/	5.978
149[c]	Madecassic acid-24-O-glucoheptonic acid	/	2.011
150[c]	Rotundic acid-24-O-glucoheptonic acid	/	3.787
151[c]	Asiatic acid-24-galloyl-28-glucose	/	6.213
152[a]	1,6-O-galloyl-2-O-cinnamoyl-glucose [19]	2.01	1.847
153[c]	Terminolic acid-24-O-glucoheptonic acid	/	2.011
154[c]	Asiatic acid-24-O-glucoheptonic acid	/	4.098
155[c]	Arjunolic acid-24-galloyl-28-glucose	/	6.213
156[c]	Arjunolic acid-24-O-glucoheptonic acid	/	4.098
157[a]	1,2,3-Tri-O-galloyl-6-O-cinnamoyl-β- D-glucose [19]	2.97	2.980
158[a]	Arjungenin [45]	4.44	4.517
159[a]	Madecassic acid [29]	4.16	3.975
160[a]	23-galloyl-arjunolic acid [58]	6.42	7.541
161[a]	Terminolic acid [56]	5.46	6.062
162[b]	Rotundic acid [59]	5.07	5.751
163[a]	Asiatic acid [30]	5.32	6.062
164[a]	Arjunolic acid [21]	5.46	6.062
